# CRISPR/Cas9 mediated knockout of the *abdominal-A* homeotic gene in fall armyworm moth (*Spodoptera frugiperda*)

**DOI:** 10.1371/journal.pone.0208647

**Published:** 2018-12-06

**Authors:** Ke Wu, Paul D. Shirk, Caitlin E. Taylor, Richard B. Furlong, Bryce D. Shirk, Daniele H. Pinheiro, Blair D. Siegfried

**Affiliations:** 1 Department of Entomology and Nematology, University of Florida, Gainesville, FL, United States of America; 2 USDA-ARS, CMAVE-IBBRU, Gainesville, FL, United States of America; West China Hospital, Sichuan University, CHINA

## Abstract

The fall armyworm (FAW), *Spodoptera frugiperda* (J.E. Smith), is an important pest of maize in the Americas and has recently been introduced into Africa. Fall armyworm populations have developed resistance to control strategies that depend on insecticides and transgenic plants expressing *Bacillus thuringiensis* toxins. The study of various resistance mechanisms at the molecular level and the development novel control strategies have been hampered by a lack of functional genomic tools such as gene editing in this pest. In the current study, we explored the possibility of using the CRISPR/Cas9 system to modify the genome of FAW. We first identified and characterized the *abdominal-A* (*Sfabd-A*) gene of FAW. *Sfabd-A* single guide RNA (sgRNA) and Cas9 protein were then injected into 244 embryos of FAW. Sixty-two embryos injected with *Sfabd-A* sgRNA hatched. Of these hatched embryos, twelve developed into larvae that displayed typical aba-A mutant phenotypes such as fused segments. Of the twelve mutant larvae, three and five eventually developed into female and male moths, respectively. Most mutant moths were sterile, and one female produced a few unviable eggs when it was outcrossed to a wild-type male. Genotyping of 20 unhatched *Sfabd-A* sgRNA-injected embryos and 42 moths that developed from *Sfabd-A* sgRNA-injected embryos showed that 100% of the unhatched embryos and 50% of the moths contained indel mutations at the *Sfabd-A* genomic locus near the guide RNA target site. These results suggest that the CRISPR/Cas9 system is highly efficient in editing FAW genome. Importantly, this gene editing technology can be used to validate gene function to facilitate an understanding of the resistance mechanism and lead to the development of novel pest management approaches.

## Introduction

The fall armyworm (FAW), *Spodoptera frugiperda* (J.E. Smith; Lepidoptera: Noctuidae), is a highly polyphagous pest that can feed on over 80 host plants, including commercially important crops such as corn (*Zea mays* L.), cotton (*Gossypium* spp. L.), and rice (*Oryza sativa* L.). Native to the Americas, this multivoltine pest has recently been introduced into and spread throughout sub-Saharan Africa, causing extensive damage to crops such as maize and sorghum and threatening the food security of many smallholder farm families [1].

*Spodoptera frugiperda* infestations have been traditionally controlled by insecticides including carbamates, pyrethroids and organophosphates but resistance to these pesticides has been widely reported [2, 3]. A newer approach that is based on the use of transgenic maize expressing the bacterium *Bacillus thuringiensis* (Bt) proteins (e.g. Cry1F) has been employed to control the pest. However, many cases of resistance to Cry1F maize have been reported in FAW field populations. First reported in Puerto Rico, resistance has been documented in Brazil, Argentina, and the southeast region of the U.S. mainland [4–8]. Thus, the emergence of Bt-resistant FAW populations poses the most serious threat to the effectiveness of using Bt crops in controlling the pest and has prompted intense interest in studying the resistance mechanism and developing novel control strategies.

Two recent studies showed that field-evolved resistance to Cry1Fa Bt corn in Puerto Rico is associated with the loss-of-function mutations in an ATP Binding Cassette subfamily C2 (ABCC2) gene [9, 10]. The *in vivo* functional confirmation of the involvement of various gene mutations (e.g. ABCC2 mutations) linked to resistance often requires creating and evaluating these mutations in a wild-type genetic background. However, such confirmation has been difficult in FAW due to a lack of functional genomic tools such as RNAi or genome editing techniques [11, 12].

The CRISPR/Cas9 (clustered regularly interspaced short palindromic repeats/(CRISPR)-associated protein 9) system is a novel genome-editing tool with advantages over previous techniques such as zinc finger nucleases and transcription activator-like effecter nucleases that are more time consuming and labor intensive [13–17]. The CRISPR/Cas9 system cleaves a specific sequence using the Cas9 nuclease coupled with a single guide RNA (sgRNA), inducing double‐stranded DNA breaks at target genomic loci that are repaired either by non‐homologous end joining or by homologous recombination in the presence of homologous donor template DNA [18]. This gene editing system has been used to precisely edit genomes of numerous eukaryotic organisms [19–23], including insect orders of Coleoptera [24], Diptera [25, 26], Lepidoptera [5, 27–31], and Hemiptera [32]. Thus, this gene editing technique facilitates functional genomic studies of model and non-model species and the development of novel genetics-based pest control approaches (for review, see [33]).

Many genes have been considered as possible targets for genetic control of pests, including homeotic genes such as *abdominal-A* (*abd-A*) [5, 27, 33]. The conserved *abdominal-A* gene is a member of *Drosophila* bithorax complex, and similar to other homeotic genes, it encodes a transcription factor that interacts with a large number of downstream target genes [34]. Amorphic (or null) alleles of *abd-A* are lethal [35, 36]. However, hemizygous viable *abd-A* alleles allow the study of many physiological roles this gene plays during the development of *Drosophila* and other insects. These include the determination of abdominal segment identities, the differentiation of the anterior body and rear somite axis, cardiac tube formation and the determination of heart cell fate in the dorsal vessel, the development of the nervous system and fat body, gonadogenesis, midgut formation and muscle patterning [35, 37–44]. Products of *abd-A* are also involved in larva-to-pupa metamorphosis in silkworm (*Bombyx mori*) [45] and the development of ventral appendages (prolegs and legs) in late-stage silkworm embryos [46].

The CRISPR/Cas9 system has been used to edit the sequence of *abd-A* in two Lepidopteran species, *Spodoptera litura* and *Plutella xylostella* [5, 27]. Mutagenesis of *abd-A in S*. *litura* and *P*. *xylostella* produces many deleterious phenotypes such as malformed segments, abnormal prolegs, anomalous gonads, and embryonic lethality [5, 27]. It is unclear whether the CRISPR/Cas9 system can be used to perform gene editing in FAW and what phenotypes would be produced when *abd-A* is mutated in this pest.

In the current study, we identified an *abd-A* gene (*Sfabd-A*) in a FAW genome and four *Sfabd-A* transcript variants in a FAW transcriptome. We used the CRISPR/Cas9 system to produce loss-of-function *abd-A* mutants and characterized their phenotypes. The CRISPR/Cas9 system was efficient in producing *abd-A* mutants in FAW. Severe abdominal morphological defects and significant lethality resulted from the disruption of the gene. These results demonstrate the potential of using the CRISPR/Cas9 system for further gene function studies and developing new approaches for genetic control of fall armyworm.

## Materials and methods

### Insect strains and rearing

Larvae of *S*. *frugiperda* corn strain were maintained on Multiple Species Artificial Diet (Southland Products, Inc.) and held at 26°C and a relative humidity (RH) of 80%, under a 16L:8D photoperiod. The adults were fed with 50% honey and kept at 26°C and an RH of 80%.

### Identification, annotation, and phylogenetic analysis of *Sfabd-A* gene

To identify putative *Sfabd-A* transcripts, tBLASTn searches using the protein sequences of *abd-A* from *D*. *melanogaster* and *B*. *mori* as queries, were performed on a FAW transcriptome that was assembled from Illumina sequencing of mRNA from the midgut tissues of FAW adults (B.D. Siegfried, unpublished).

The initial annotations of *Sfabd-A* transcripts were performed based on their homology with well-studied query sequences from *D*. *melanogaster*. These annotations were further verified by performing reciprocal BLASTp searches, using the deduced amino acid sequences of *Sfabd-A* isoforms against databases from which query sequences were derived. For further validation, the deduced amino acid sequences of these transcripts were used to search the conserved domain database (CDD: http://www.ncbi.nlm.nih.gov/Structure/cdd/wrpsb.cgi) to ascertain that they contain the canonical domains of *abd-A* protein (Domain IDs: pfam00046 and cl27820). The nucleotide and deduced amino acid sequences of *Sfabd-A* transcripts were deposited in GenBank.

The *Sfabd-A* genomic sequence was identified in a sequenced FAW genome (rice variant assembly v1.0 deposited at https://bipaa.genouest.org/is/) after performing BLAST searches using the nucleotide sequences of *Sfabd-A* transcripts. The exon/intron boundary of *Sfabd-A* gene was determined by aligning *Sfabd-A* transcript sequences and genomic sequence using Splign (https://www.ncbi.nlm.nih.gov/sutils/splign/splign.cgi?textpage=online&level=form).

To perform a phylogenetic analysis of *abd-A*, the deduced amino acid sequences of *Sfabd-A* isoforms were aligned with those of corresponding orthologs from 13 species (S1 Table) using the MUSCLE alignment algorithm [47]. A midpoint-rooted tree was constructed using a maximum likelihood approach with PhyML3.0. [48].

### CRISPR/Cas9-mediated gene editing

sgRNA candidates targeting the exon I region of *Sfabd-A* gene were designed using a sgRNA designer website hosted by the Broad Institute (https://portals.broadinstitute.org/gpp/public/analysis-tools/sgrna-design). Several sgRNA candidates that were highly ranked based on estimated on-target activities were further evaluated for potential off-target effect by the use of BLAST searches applied to the *S*. *frugiperda* genome (https://bipaa.genouest.org/is/) and a visual analysis of the results. Candidate sgRNAs that produced genomic hits, other than *Sfabd-A*, which perfectly matched the final 12 nucleotides of the target sequence and NGG PAM (protospacer adjacent motif) sequence were discarded [19]. One candidate that targeted nucleotide 671 to 691 of exon I was chosen for the current study (S1 Fig). A control sgRNA targeting *eGFP* gene was selected based a previous study [5].

sgRNAs were produced using a method modified from that of Bassett et al. [25]. Briefly, sgRNA templates were made by amplifying overlapping forward and reverse primers containing a T7 promoter and stem loop structures needed for Cas9 binding (see S2 Table for primer sequences) with Taq polymerase (Fisher Scientific) in 50-μl reactions containing 50 mM of Tris (pH9.2), 16 mM of ammonium sulfate, 1.75 mM of MgCl_2_, 350 nM of each dNTP, and 0.5 μM of forward and reverse primers. The PCR was carried out using three linked profiles in a C1000 Touch Thermal Cycler (Bio-Rad): (1) one cycle consisting of denaturation at 98°C for 30 s; (2) thirty-five cycles each consisting of denaturation at 98°C for 30 s, annealing at 60°C for 30 s and extension at 68°C for 15 s; (3) one cycle consisting of polishing at 68°C for 10 min. After amplification, PCR products were analyzed on 2% agarose gels containing ethidium bromide and bands of expected size (125 bp) were excised and purified using a Gel Extraction kit (Qiagen) according to the manufacturer’s protocol. sgRNAs were synthesized (4 h at 37°C) from 800 ng of purified PCR products using a MEGAscript T7 kit (cat. No. AM1626, Life technologies) and purified according to manufacturer’s instructions. Concentrations of purified sgRNAs were determined by spectrophotometry (NanoDrop 1000, Thermo Scientific). Purified sgRNAs were stored in elution buffer at −80°C until further use.

### Embryo microinjection

FAW females were caged to allow oviposition on paper towels, which were renewed before the beginning of the dark cycle. Embryos were aligned in single rows on cellophane with the micropyle up and microinjected with sgRNA (e.g. at 250 ng/μl) along with Cas9 protein (e.g. at 500 ng/μl; Cat. No. 1081058, IDT) into the medial line of each embryo within four hours of oviposition. After injection, embryos were incubated in a humidified chamber (RH ~ 80%) at 26°C for three days until hatching.

### Genomic DNA extraction and identification of *Sfabd-A* mutations

The genomic DNA of unhatched embryos was extracted using a method adapted from that of Wu and Hoy [49]. Briefly, individual unhatched embryos were homogenized in 30 μl of cell lysis buffer (Qiagen) using a motorized microcentrifuge tube pellet pestle (Fisher Scientific) for 20 s on ice. Residual sample material on the pestle was rinsed off with 70 μl of cell lysis buffer and pooled with that in the microcentrifuge tube. Pooled samples were then incubated at 65°C for 15 min, after which time each sample was chilled on ice for 1 min, and then 33 μl of precipitation buffer (Qiagen) was added. After vortexing for 20 s, samples were incubated on ice for 5 min. Following 10 min of centrifugation at 14,000 rpm at 4°C, supernatants were transferred to a new 1.5 ml microcentrifuge tube containing 15 μg of GlycoBlue (Ambion). After a brief vortex, 100 μl of isopropanol was added to each sample. Samples were then incubated for 1 h at −80°C after mixing by gently inverting for 3 times. Genomic DNA was recovered by centrifugation at 14,000 rpm for 30 min at 4°C. Precipitates were rinsed with 75% ethanol before being dried on bench for 10 min. Genomic DNA was then dissolved in 20 μl of TE (10 mM Tris, 1 mM EDTA, pH 7.5) for 1 h at room temperature and stored at −20°C until further assays.

Moth genomic DNA was extracted from the hind legs using a method adapted from that provided by DNeasy kit (Qiagen). Briefly, each sample was repeatedly (2–3 times) homogenized by hand using a disposable pestle in a 1.5 ml tube after it was frozen in liquid nitrogen. After placing the sample on ice, 180 μl of ATL buffer and 20 μl of proteinase K solution were added. The sample was then incubated at 56°C for 10 min. After vortexing the sample for 15 s, 200 μl of AL buffer was added, followed by incubation at 56°C for 10 min. Following 3 min of centrifugation at 14,000 rpm at 25°C, the supernatant was transferred to a new 1.5 ml tube. Two hundred μl of 100% ethanol was added to the supernatant and the mixture was added to a DNeasy Mini spin column sitting on top a 1.5 ml collection tube. The genomic DNA was retained on the column after a 1 min centrifugation at 14,000 rpm. The column was then washed with 500 μl of AW1 and AW2 buffer. The spin column was transferred to a new 1.5 ml tube and 50 μl of AE buffer was added. After incubation at 25°C for 1 min, the genomic DNA was recovered by a 1 min centrifugation at 14,000 rpm. Concentrations of purified genomic DNA were determined by spectrophotometry (NanoDrop 1000, Thermo Scientific). Purified genomic DNA was stored at −20°C until further use.

High resolution melt analysis (HRMA) and sequencing were used to genotype potential *Sfabd-A* mutations in *Sfabd-A* sgRNA-treated embryos and moths. Primers (S2 Table) were designed to produce a 179 bp product spanning the presumed Cas9 cleavage site with the use of Primer-BLAST (https://www.ncbi.nlm.nih.gov/tools/primer-blast/). The PCR reactions were performed with 5 μl of the Precision Melt Supermix for HRM analysis (Bio-Rad), 0.5 μl of each primer (20 μM), 0.5 μl of genomic DNA (10 ng/ μl), and water up to 10 μl. The PCR was carried out in a CFX96 Real-Time System (Bio-Rad) using white 96 well plates with four linked profiles: (1) one cycle consisting of denaturation at 95°C for 2 min; (2) forty cycles each consisting of denaturation at 95°C for 10 s, annealing and extension at 60°C for 30 s; (3) one cycle consisting denaturation at 95°C for 60 s and annealing at 60°C for 60 s; (4) one cycle of melting with temperature increasing by 0.02°C/s until 95°C for 10 s, then cooling at 40°C. Melt curves were analyzed using Precision Melt Analysis software provided by Bio-Rad. PCR products synthesized for HRMA were cloned into pJET1.2/blunt vector (ThermoFisher) and sequenced by GENEWIZ (South Plainfield, NJ, USA) using primers designed for the HRMA.

### Statistical analysis

Pairwise comparisons of each pair of treatment were performed using Fisher’s exact test (JMP Pro 13; SAS Institute, Cary, NC).

## Results

### Identification and characterization of *Sfabd-A* gene

**Fig 1:**
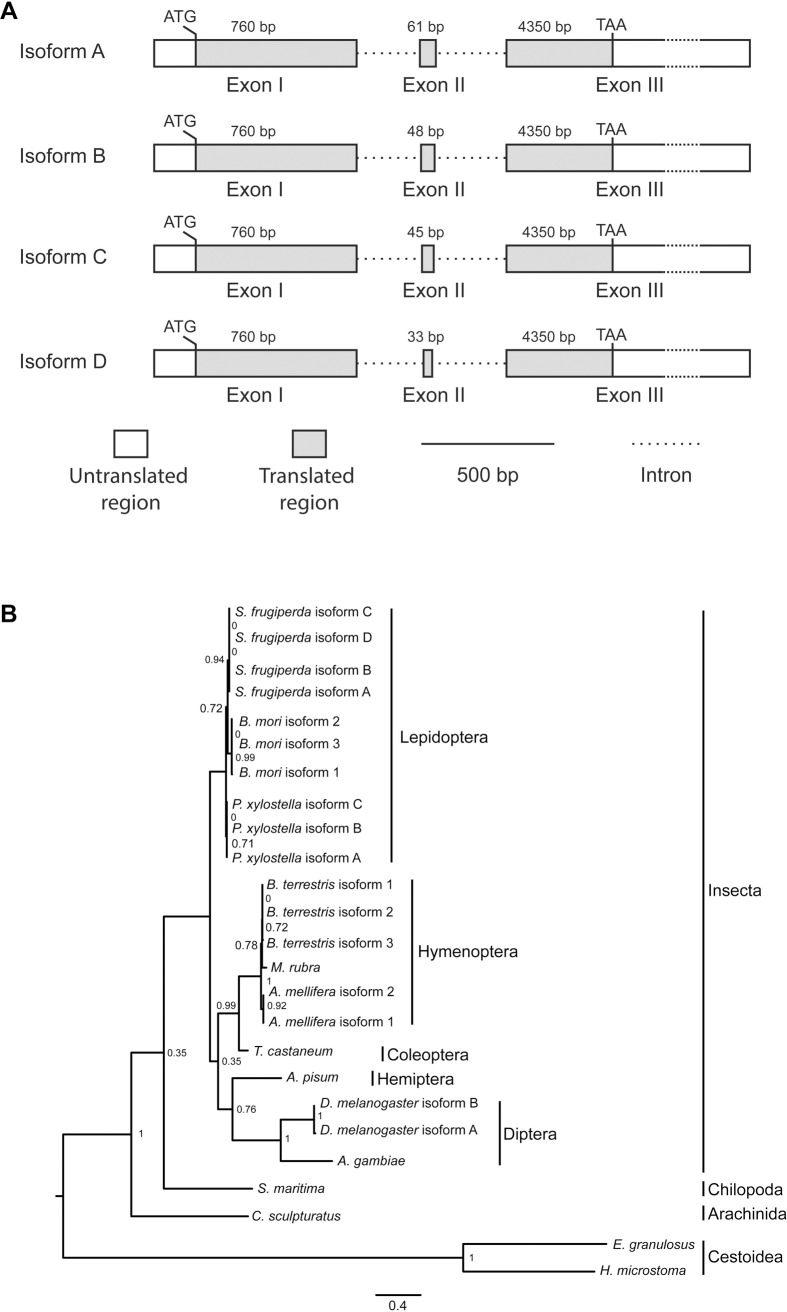
Transcript structure of *Sfabd-A* and phylogenetic tree of *abd-A* of 14 select species. **(A)** Schematic structures of four (A-D) *Sfabd-A* transcript isoforms. Numbers refer to the length in base-pairs (bp) of each gene component. ATG and TAA denote the translation initiation and termination codons, respectively. **(B)** A midpoint-rooted phylogenetic tree based on the alignment of the amino acid sequences of *abd-A* of 14 species was generated using a maximum likelihood approach [48]. aLRT SH-like branch support values are shown at the nodes. The scale bar represents the numbers of substitutions per site. The tree includes four major branches: Insecta, Chilopoda, Arachinida, and Cestoidea.

Annotation of *Sfabd-A* transcripts in a FAW transcriptome revealed four splice variants (A-D) with coding sequences of lengths 1035, 1047,1050, and 1062 bp, encoding 344, 348, 349, and 353 amino acids, respectively (S2 Fig). Manual annotation of the *Sfabd-A* gene in a sequenced FAW genome produced one putative full-length gene model (S1 Fig). Each splice variant of *Sfabd-A* has three exons and differs only in the size of the second exon (Fig 1A).

A phylogenetic analysis of *abd-A* of 14 select species revealed order-specific clustering within insects (Fig 1B). *Spodoptera frugiperda*, *P*. *xylostella* and *B*. *mori* are clustered into the Lepidoptera clade that is divergent from the Hymenoptera, Coleoptera, Hemiptera, and Diptera clades. These results suggest that *abd-A* likely encodes conserved functions among different insect orders.

### Phenotypes induced by the disruption of *Sfabd-A*

We initially performed a pilot study to determine whether various concentrations of sgRNAs might affect embryo hatch rate. *Sfabd-A* or *eGFP* sgRNA was injected into FAW embryos at three concentrations (i.e. 62.5 ng/μl, 125 ng/μl, and 250 ng/μl), along with Cas9 protein (i.e. 125 ng/μl, 250 ng/μl, and 500 ng/μl, respectively). *Sfabd-A* sgRNA injections at these concentrations resulted in similar embryo hatch rates, which were consistently lower than those of *eGFP* sgRNA-injected embryos (S3 Table). Because higher concentrations of sgRNAs would likely result in higher rates of mutagenesis [5, 27], all subsequent sgRNA injections were performed at concentration of 250 ng/μl.

Approximately 85% of the uninjected embryos hatched. By comparison, 67.2% of the embryos injected with *eGFP* sgRNA hatched (Table 1). The slight, but significant reduction in the hatch rate of *eGFP* sgRNA-injected embryos was likely caused by damage sustained during the injection process (Tables 1 and S4). Interestingly, the hatch rate of embryos injected with *Sfabd-A* sgRNA was significantly lower than those of uninjected and *eGFP* sgRNA controls (Tables 1 and S4), indicating that *Sfabd-A* sgRNA treatment had a deleterious effect on the viability of embryos. The unhatched embryos that received *Sfabd-A* sgRNA injection appeared discolored and lacked some of the developing organs seen in uninjected control embryos on day one and two post oviposition (S3 Fig). Importantly, the differences in hatch rates among different treatment groups were comparable to those seen in the pilot study (S3 Table).

**Table 1:** CRISPR/Cas9-mediated *Sfabd-A* mutagenesis.

Treatment	Number of embryos	Percent hatch	Percent development (larva-pupa)	Percent development (pupa-moth)	Percent G_0_ mosaic^a^
Uninjected control	120	85.8 A^b^	97.0 A	99.0 A	0 A
*eGFP* sgRNA injection	165	67.2 B	87.3 B	96.9 A	0 A
*Sfabd-A* sgRNA injection	244	25.4 C	70.9 C	95.4 A	19.3 B

^a^ Percent G_0_ mosaic = (Number of larvae showing mutant phenotype/Total number of larvae)%.

^b^ Different capital letters denote significant difference (Fisher’s exact test, details can be found in S4 Table).

**Fig 2:**
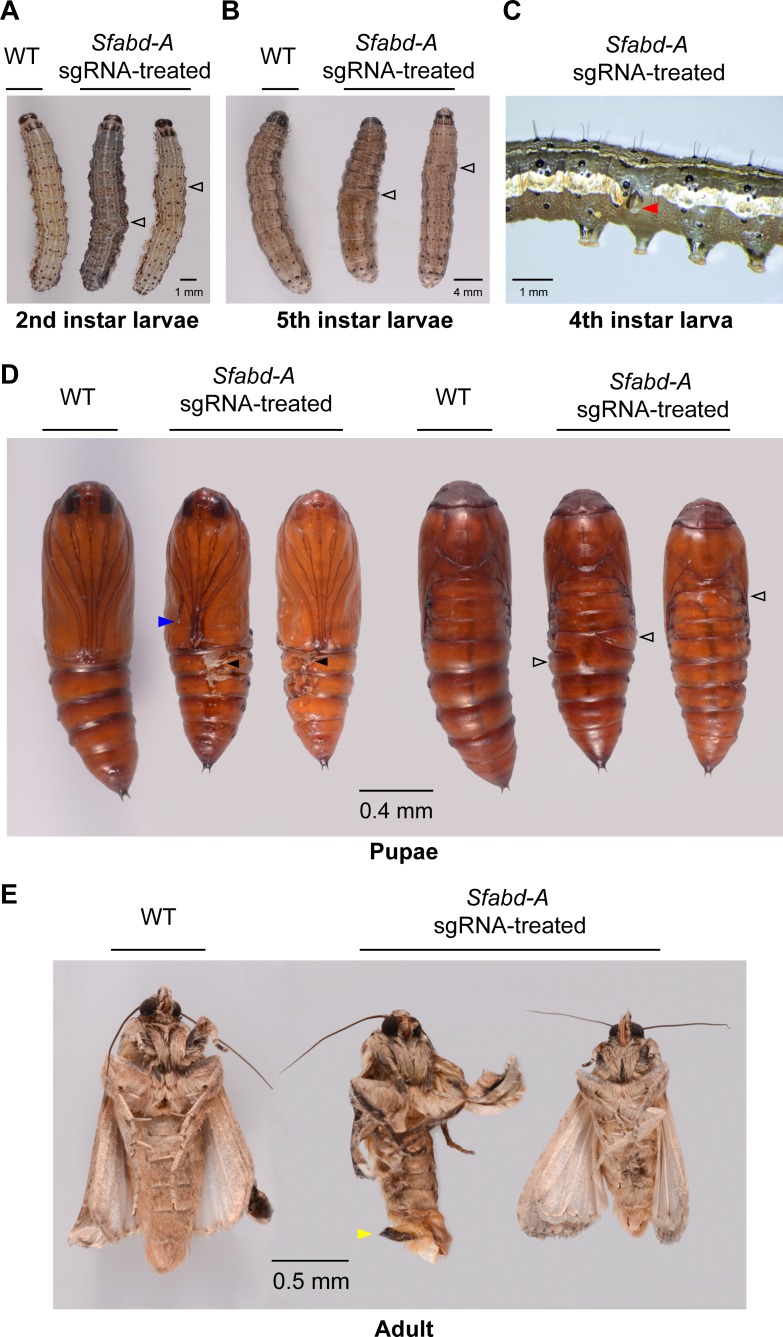
Phenotypes of *Sfabd-A* G_0_ chimeric mutants. Disruption of body segments (open black arrow heads) was observed in the 2nd **(A)** and 5th **(B)** instar larvae of *S*. *frugiperda*, respectively. WT: Wild type (*eGFP* sgRNA-treated). A malformed proleg was observed in a 4th instar larva **(C)**. Multiple morphological defects **(D)** were observed in pupae, including altered antenna formation (blue arrow head), lesions on the cuticles (black filled arrow heads), and fused segments (black open arrow heads). Two male moths were smaller than an *eGFP* sgRNA-treated male. One mutant showed malformed claspers (yellow arrow head) that were permanently open **(E)**.

In the *Sfabd-A* sgRNA treatment, 12 of the 62 hatched larvae displayed mutant phenotypes in which abdominal segments were fused or prolegs were malformed (Table 1 and Fig 2A–2C). Eight of the 62 hatched larvae died within two days and nine more died during later stages of larval development. A total of 45 larvae survived to 5th instar (Fig 2B) and all but one pupated (Fig 2D). The percent of larva that developed into pupa was lower in the *Sfabd-A* sgRNA treatment group than those in the uninjected and *eGFP* sgRNA controls (Tables 1 and S4), suggesting that *Sfabd-A* sgRNA, and to a lesser extent *eGFP* sgRNA treatment, exerted a detrimental effect during this stage of development. By comparison, neither *eGFP* sgRNA nor *Sfabd*-A sgRNA treatment appeared to affect the development of pupae into adults (Tables 1 and S4).

Ten larvae with mutant phenotypes developed into pupae that showed additional mutant phenotypes such as cuticular lesions and malformed antennae (Fig 2D). Of 44 pupae, all but two (i.e. with mutant phenotypes) developed into moths. Moths (5 males and 3 females) with mosaic mutations were shorter in length when compared to wild-type moths (Fig 2E, males shown) and one male mutant moth had malformed claspers (Fig 2E).

**Fig 3:**
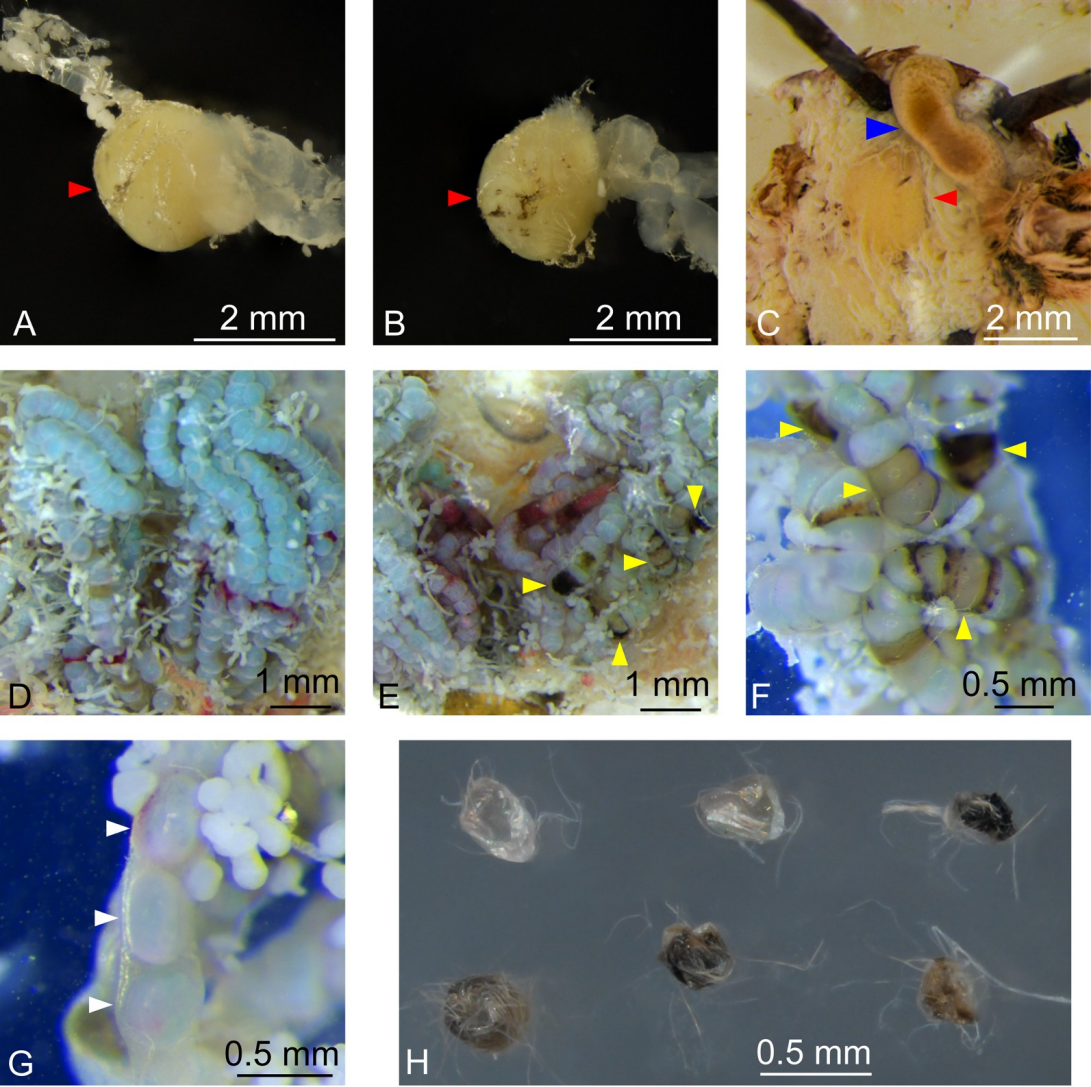
Phenotypes of *Sfabd-A* G_0_ male and female reproductive organs. A comparison of the testis (red arrow head) of a wild-type (*eGFP* sgRNA-treated) **(A)** and mutant **(B)** FAW moth showed a difference in shape. Meconium (blue arrow head) was present inside the abdomen of a mutant male moth **(C)**. A comparison of wild-type (*eGFP* sgRNA-treated) **(D)** and mutant **(E)** ovaries revealed the presence of necrotic follicles (yellow arrow heads) in the mutant ovary. **(F)** shows a more detailed picture of some of the necrotic follicles in the mutant ovary. Several reabsorbed follicles (white arrow heads) were found in the mutant ovary **(G)**. A mutant female that was outcrossed to a wild-type male produced a few malformed eggs **(H)**.

Each moth exhibiting a mutant phenotype was outcrossed to a wild-type adult. All five mutant males appeared to be sterile because no eggs were deposited by the wild-type female. Only one mutant female exhibited any evidence of oviposition although the few eggs that were deposited did not hatch (Fig 3H). One male showing cuticular lesions and malformed claspers survived for five days before being dissected to assess the status of internal organs. The testis of this male appeared semi-spheroidal when compared to the spheroidal wild-type testis (Fig 3A and 3B), indicating that mutant testis formation might be abnormal. In addition, the meconium was still retained and had not been voided after adult eclosion (Fig 3C), suggesting that there were disruptions to the normal endocrinological/behavioral activities of the pharate adult that interfered with adult eclosion as had been previously reported in *B*. *mori* [50, 51]. Similarly, a female showing cuticular lesions that survived for 10 days was dissected to evaluate the status of internal organs. Examination of ovaries revealed the presence of many necrotic follicles and some reabsorbed eggs (Fig 3D–3G), which may have resulted from disrupted egg movement as reported for the phenotypes from different mutations of *abd-A* in *Drosophila* [37].

### CRISPR/Cas9-mediated mutagenesis of *Sfabd-A*

**Fig 4:**
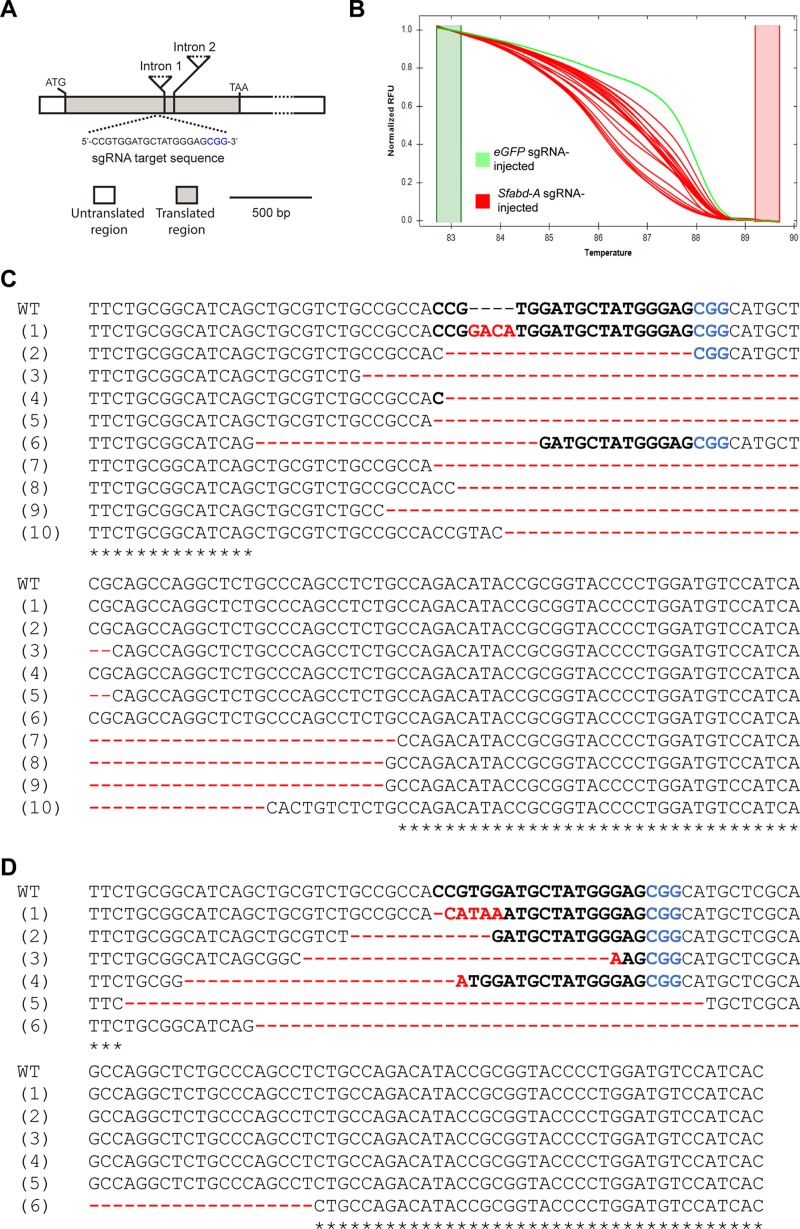
CRISPR/Cas9-mediated Sfabd-A mutations. (A) Schematic representation of the sgRNA sequence targeted in Exon I of Sfabd-A locus. (B) Difference curves generated from the melting of amplicons that were produced by amplification of a portion of the Sfabd-A gene containing the sgRNA target site in unhatched embryos. Various indel mutations were found in unhatched embryos (C) and G0 mutant moths (D). Sequences are of the sgRNA site (WT: wild type) and several induced mutations (1–10 in (C) and 1–6 in (D)). Mutations include both small deletions (-) and insertions near sgRNA target site. Sequences of the sgRNA target site are lettered in black and bold, the PAM motif in blue, and the insertions/deletions in red.

Compared to embryos injected with *eGFP* sgRNA, the majority (~75%) of embryos injected with *Sfabd*-*A* sgRNA did not hatch (Table 1), suggesting that the high mortality in these embryos may have been caused by amorphic mutations of *Sfabd-A* because a complete loss *abd-A* functions is lethal [35, 36]. Genomic DNA of 20 randomly selected unhatched embryos that were injected with *Sfabd-A* sgRNA were used as templates to perform HRMA by amplifying the region flanking the sgRNA target site (Figs 4A and S1). Compared to the melt curve that was produced by amplicons generated from control (*eGFP* sgRNA) genomic DNA, all curves produced by amplicons generated from the genomic DNA of *Sfabd-A* sgRNA-injected embryos showed a change in shape and position (Fig 4B), indicating the presence of possible mutation events [52, 53]. Cloning and sequencing of these amplicons confirmed the presence of indel mutations in all embryos injected with *Sfabd-A* sgRNA (Figs 4C and S4 and Table 2). Notably, when multiple clones derived from the amplicons of an unhatched embryo were sequenced, various deletion mutations (E13_1—E13_3) and wild-type *Sfabd-A* sequence (E13_4) were found (S4 Fig). By comparison, thirteen embryos that were injected with *eGFP* sgRNA were screened and found to contain wild-type copies *Sfabd-A* gene (Table 2 and S4 Fig).

**Table 2:** Mutagenesis rates of CRISPR/Cas9-mediated *Sfabd-A* gene editing.

Treatment	Number of unhatched embryos screened for mutant genotype^a^	Percent mutation of unhatched embryos	Number of moths screened for mutant genotype	Percent mutation of moths
Uninjected control	0	N.A.	4	0
*eGFP* sgRNA injection	13	0	2	0
*Sfabd-A* sgRNA injection	20	100	42	50

^a^ Screening of mutant genotype was performed using high resolution melt analysis and sequencing.

HRMA of amplicons produced from the genomic DNA of all of the moths (42) that developed from *Sfabd-A* sgRNA-injected embryos demonstrated that 50% of these moths contained mutated *Sfabd-A* sequences (S1 File). Sequencing of a subset of these amplicons confirmed the presence of various deletion mutations of *Sfabd-A* gene in these moths (Figs 4D and S4 and S1 File). And similar to the results obtained when an unhatched *Sfabd-A* egg was evaluated in detail (see above), different deletion mutations (BT_1—BT_6) and wild-type *Sfabd-A* sequence (BT_7) were found when several clones derived from the amplicons of a mutant moth were sequenced (S4 Fig).

Considering that unhatched and hatched embryos made up ~75% and ~25% of the total number of embryos, respectively, we estimate the mutagenesis rate for all embryos injected with *Sfabd-A* sgRNA to be approximately 87.5% (i.e. (75% × 100%) + (25% × 50%)). Thus, the CRISPR/Cas9 system was effective and efficient in inducing mutagenesis at the *Sfabd-A* locus in FAW genome.

## Discussion

The current study demonstrates that the CRISPR/Cas9 system efficiently created indel mutations in *abd-A* gene of FAW. The estimated G_0_ mutagenesis rate (87.5%) is comparable to those (88%-100%) achieved in *P*. *xylostella*, silkworm, *Helicoverpa zea* (Boddie), and *Helicoverpa armigera* using a similar system [5, 29–31]. Thus, our data suggest that the CRISPR/Cas9 system can be used as a powerful tool to edit the genome of *S*. *frugiperda*.

The majority of the embryos injected with *Sfabd-A* sgRNA did not hatch (Table 1), suggesting that indel mutations in these embryos were likely amorphic and resulted in a complete loss of *abd-A* function, which has been shown to cause embryonic lethality in *Drosophila* [35, 36]. Those embryos that hatched likely experienced only a partial loss of *abd-A* function or were not transformed. Many mosaic mutant larvae displayed fusion of abdominal segments and proleg abnormalities, similar to what was observed when *abd-A* was mutated in *P*. *xylostella* [5]. These results suggest that *Sfabd-A* is required for the formation of segments and prolegs in *S*. *frugiperda*.

A G_0_ mutant male moths displayed abnormal external and internal genitalia, and a mutant female moth showed necrotic follicles (Figs 2 and 3). These results are consistent with the notion that *abd-A* is required for the development of gonads as seen in *Drosophila* and *P*. *xylostella* [5, 37, 38]. The abnormal gonads may have contributed to the sterility of males and females observed in the current study.

In summary, the wide range of defects observed in the current study were similar to those seen in other insects when different *abd-A* mutant alleles were produced and were likely caused by distinct *Sfabd-A* mutations created by the CRISPR/Cas9 system [5, 27, 36, 37, 46]. Clearly, further studies are needed to confirm and elucidate the details of the causal relationship between various *Sfabd-A* mutations and different phenotypes seen in this study. Similarly, additional studies are also required to reveal the exact role each *Sfabd-A* isoform plays in FAW.

Unfortunately, the sterility in G_0_ moths and a lack of viable G_1_ eggs preclude the assessment of germline transmission of *Sfabd-A* mutations. Future studies of gene mutations that do not result in sterility will help evaluate the germline mutation in *S*. *frugiperda* mediated by the CRISPR/Cas9 system. Similarly, further studies are needed to investigate whether the CRISPR/Cas9 system can be used to edit other genomic loci with efficiencies similar to those achieved in the current study and whether this genome editing technology can be used to create knock-in mutations in FAW based on homology-directed repair.

The mutagenesis rate (50%) in moths is higher than the mosaic rate (~19%) (Tables 1 and 2). Several possibilities may account for this discrepancy. It is possible that CRISPR/Cas9-mediated events occurred in a subset of cells in some embryos. In these cases, mutant phenotype would only be produced in larvae that derived from embryos in which CRISPR/Cas9-generated indels occurred in progenitor cells that later developed into the abdominal segments and genitalia. It is also possible that CRISPR/Cas9-mediated events created an array of heterozygotic or heteroallelic indels within the same cells in some embryos. Hence, it is important to point out that the mosaic rate likely reflects the net result of a multitude of varied mutations (S4 Fig). Further studies are needed to evaluate the precise nature of CRISPR/Cas9-mediated mutation events in FAW.

In cases where heterozygotic mutations were made, both copies of *abd-A* may be needed to be mutated to produce a robust mutant phenotype in fall armyworm. A recently developed technique (i.e. mutagenic chain reaction) that is based on the CRISPR/Cas9 system may be employed to convert these potential heterozygous mutations to homozygous ones [54]. This technique may also provide a potent gene drive system for the delivery of transgenes in pest populations [54]. Utilization of gene drive replacement to restore sensitivity may be a viable control approach as FAW populations have developed resistance to multiple classes of insecticides and Bt corn [2–8]. The CRISPR/Cas9 system has the potential to accelerate the study of resistance mechanisms and the development of innovative genetics-based control strategy.

## Supporting information

S1 Table *abd-A* genes used in a phylogenetic analysis. (DOCX)

S2 Table Primers used in the current study. (DOCX)

S3 Table Hatch rates of fall armyworm embryos injected with eGFP or Sfabd-A sgRNA at various concentrations. (DOCX)

S4 Table Pairwise comparisons of each pair of treatment. (DOCX)

S1 Fig The annotated genomic sequence of *Sfabd-A* gene. (DOCX)

S2 Fig The nucleotide and amino acid sequences of *Sfabd-A* isoforms. (TXT)

S3 Fig Representative pictures of uninjected and *Sfabd-A* sgRNA-injected FAW embryos. (DOCX)

S4 Fig Representative nucleotide sequences spanning the *Sfabd-A* sgRNA target region of samples included in the current study. (TXT)

S1 File HRMA data included in the current study. (XLSX)
