# Supplementary material for: CRISPR/Cas9 mediated knockout of the abdominal-A homeotic gene in fall armyworm moth (Spodoptera frugiperda)
Source: PLoS One. 2018 Dec 6;13(12):e0208647. doi: 10.1371/journal.pone.0208647 (PMC6283638; doi:10.1371/journal.pone.0208647)
Supplement: S1 Table — (DOCX) [file pone.0208647.s001.docx]

**S1 Table**. *abd-A* genes used in a phylogenetic analysis.

| Species | Name in tree | GenBank accession ID |
| --- | --- | --- |
| *Spodoptera frugiperda* | *S. frugiperda* isoform A | MH541833 |
|  | *S. frugiperda* isoform B | MH541834 |
|  | *S. frugiperda* isoform C | MH541835 |
|  | *S. frugiperda* isoform D | MH541836 |
| *Bombyx mori* | *B. mori*  isoform 1 | ACD10794.1 |
|  | *B. mori*  isoform 2 | ABY66346.1 |
|  | *B. mori*  isoform 3 | NP_001166809.1 |
| *Plutella xylostella* | *P. xylostella* isoform A | XP_011569267.1 |
|  | *P. xylostella* isoform B | XP_011569268.1 |
|  | *P. xylostella* isoform C | XP_011569269.1 |
| *Bombus terrestris* | *B. terrestris* isoform 1 | XP_003402189.1 |
|  | *B. terrestris* isoform 2 | XP_003402190.1 |
|  | *B. terrestris* isoform 3 | XP_003402191.1 |
| *Myrmica rubra* | *M. rubra* | AAK06846.2 |
| *Apis mellifera* | *A. mellifera* isoform 1 | XP_016772643.1 |
|  | *A. mellifera* isoform 2 | XP_394120.4 |
| *Tribolium castaneum* | *T. castaneum* | NP_001034518.1 |
| *Acyrthosiphon pisum* | *A. pisum* | XP_001944629.2 |
| *Drosophila melanogaster* | *D. melanogaster* isoform A | NP_476693.1 |
|  | *D. melanogaster* isoform B | NP_732176.1 |
| *Anopheles gambiae* | *A. gambiae* | XP_003436886.1 |
| *Strigamia maritima* | *S*. *maritima* | ABD16213.1 |
| *Centruroides sculpturatus* | *C. sculpturatus* | XP_023228869.1 |
| *Echinococcus granulosus* | *E. granulosus* | EUB63688.1 |
| *Hymenolepis microstoma* | *H. microstoma* | CDS32025.2 |
