# Supplementary material for: CRISPR/Cas9 mediated knockout of the abdominal-A homeotic gene in fall armyworm moth (Spodoptera frugiperda)
Source: PLoS One. 2018 Dec 6;13(12):e0208647. doi: 10.1371/journal.pone.0208647 (PMC6283638; doi:10.1371/journal.pone.0208647)
Supplement: S2 Table — (DOCX) [file pone.0208647.s002.docx]

**S2 Table**. Primers used in the current study.

| Primer name | Sequence (5’-3’) |
| --- | --- |
|  | sgRNA synthesis |
| sgRNA-Rev | AAAAGCACCGACTCGGTGCCACTTTTTCAAGTTGATAACGGACTAGCCTTATTTTAACTTGCTATTTCTAGCTCTAAAAC |
| *Sfabd-A* sgRNA-F | GAAATTAATACGACTCACTATAGGCGCTCCCATAGCATCCACGGGTTTTAGAGCTAGAAATAGC |
| *eGFP*-sgRNA-F | GAAATTAATACGACTCACTATAGGGGCGAGGGCGATGCCACCTAGTTTTAGAGCTAGAAATAGC |
|  | HRMA and cloning/sequencing of *Sfabd-A* genomic fragment flanking the sgRNA target region |
| *Sfabd*-*A* F | GGTGTCGGCAGCATCGG |
| *Sfabd*-*A* R | CAGTGATGGACATCCAGGGGTA |
