# Supplementary material for: CRISPR/Cas9 mediated knockout of the abdominal-A homeotic gene in fall armyworm moth (Spodoptera frugiperda)
Source: PLoS One. 2018 Dec 6;13(12):e0208647. doi: 10.1371/journal.pone.0208647 (PMC6283638; doi:10.1371/journal.pone.0208647)
Supplement: S3 Table — (DOCX) [file pone.0208647.s003.docx]

**S3 Table**. Hatch rates of fall armyworm embryos injected with *eGFP* or *Sfabd-A* sgRNA at various concentrations.

| Treatment | No. of embryos | No. of embryos that hatched | Percent hatch |
| --- | --- | --- | --- |
| Uninjected | 150 | 64 | 42.6 |
| *eGFP* sgRNA at 62.5 ng/µl | 50 | 13 | 26 |
| *eGFP* sgRNA at 125 ng/µl | 50 | 12 | 24 |
| *eGFP* sgRNA at 250 ng/µl | 50 | 15 | 30 |
| *Sfabd-A* sgRNA at 62.5 ng/µl | 100 | 7 | 7 |
| *Sfabd-A* sgRNA at 125 ng/µl | 100 | 8 | 8 |
| *Sfabd-A* sgRNA at 250 ng/µl | 100 | 8 | 8 |
