# Supplementary material for: CRISPR/Cas9 mediated knockout of the abdominal-A homeotic gene in fall armyworm moth (Spodoptera frugiperda)
Source: PLoS One. 2018 Dec 6;13(12):e0208647. doi: 10.1371/journal.pone.0208647 (PMC6283638; doi:10.1371/journal.pone.0208647)
Supplement: S4 Table — (DOCX) [file pone.0208647.s004.docx]

**S4 Table**. Pairwise comparisons of each pair of treatment.

| Comparison | Fisher’s exact p-value (2-Tail) | | | |
| --- | --- | --- | --- | --- |
|  | Percent hatch | Percent development (larva-pupa) | Percent development (pupa-moth) | Percent G_0_ mosaic |
| Uninjected vs *eGFP* sgRNA | 0.0003 | 0.0104 | 0.3636 | Not applicable |
| Uninjected vs *Sfabd-A* sgRNA | <0.0001 | <0.0001 | 0.2213 | <0.0001 |
| *eGFP* sgRNA vs *Sfabd-A* sgRNA | <0.0001 | 0.0133 | 0.6473 | <0.0001 |
