# Supplementary material for: CRISPR/Cas9 mediated knockout of the abdominal-A homeotic gene in fall armyworm moth (Spodoptera frugiperda)
Source: PLoS One. 2018 Dec 6;13(12):e0208647. doi: 10.1371/journal.pone.0208647 (PMC6283638; doi:10.1371/journal.pone.0208647)
Supplement: S1 Fig — (DOCX) [file pone.0208647.s005.docx]

**S1 Fig.**

The annotated genomic sequence of *Sfabd-A* gene**.** Exons (*Sfabd-A* isoform A) are in upper case letters. Open reading frames are in bold and italicized letters. Introns are in lower case letters. The sgRNA target sequence is underlined. Oligonucleotides used to perform HRMA analysis are in red. The sequence was manually annotated based on an alignment of *Sfabd-A* mRNA and genomic sequences (entry SFRU_RICE_001372 of the rice variant-assembly v1.0) deposited on <https://bipaa.genouest.org/is/>. The lengths of the *Sfabd-A* gene, mRNA and ORF are 41,715 bp, 5170 bp, and 1062 bp, respectively.

GCCACGACGGGGAATTCGGAGTCGTAGAGCCTCATACGCGGGTCATAAACGATCCGCGGCCATAGTAGAGGTGCACGCGCGAGAATCGATCCGAATCGCCGGCGATAAACGCATCTCCCGCGAGGCGATCGTCGCGCGCCGCGTCGCCGCCGAGCG***ATGAGTTCCAAGTTCATCATCGATAGCATGCTCCCGAAGTACCACCAGCAGTTCCACCACCAGAATTTGTTCGCGGGAACTGGGGCGTCGCCTATAGAGGCGTCGCTGTCGTCTTCGCTGTCGTCGTCTCTGTCGACGTCGCTTTCGAGTTCGCTGTCTGGCGGACTGGGAGCGGCAGCGCTGGGGGCAGGCTCGCCGGGCGCCGGCAGCCCGCAGCGGTCCTCATCCTCATCATCAGCATCGCCGGGCGCACCGGCGAGGATGTATCCTTACGTGTCGCATCACCAGCAGTTCGGAGGTTCGGTGCCGTTCTCTGCTGGTGGCGGTTTGTCCGCGGCGGATGACAAGAGTTGTCGTTACCCGACGGCTGTGGGAGGAGACCCAATGGTGAACTACGCGCTGGGCCAGCACAACGGTGGAGCAGCGGTGTCGGCAGCATCGGCCAGCATGGCAGCCGCTGCACAGTTTTATCATCAAGCAGCAGCCTCTGCGGCATCAGCTGCGTCTGCCGCCACCGTGGATGCTATGGGAGCGGCATGCTCGCAGCCAGGCTCTGCCCAGCCTCTGCCAGACATACCGCGGTACCCCTGGATGTCCATCACTG***gtaaatgttttactccttcactgcatgatcgtttgcgactttgccgtgtctgaaacaattgttatgtattagatattttttgttgcttattaaagtataacaaaagtttccttaggaagacttagtacataggttgttatgtactttgctattaaattggccttaatgttttcgttttgtatggtaatgttttagacgtataaaatagaatttatttattattttgccgctttagaagatcatttttcgttcggctttatggttaacttttcatttatcaagaacatccaatttatcaagatatgcttttagttaattgaatactatttagttacctaatacgtaattatttgacaaaatagttataaaaaaaacatataaaaggaaacaacaaagacaatattttagtaaccatgacgtgatgttccatttagaaaaaagcattcaacctatttcttgcaaattaaaaccatttgatactattttagcacttatgtcccaataaaataattcaggcagtgcaatataaatgcaataaaaccgagcattattatttttactatcaataaatcagaacaagagtcctttacgggtgtgaaataactttgacagaagttagcagcaattttaataaatactagccataaaataaaatcaaaacatatgtaatttgttgtggtgaattgaaataaggtaattaaataaataataagggtgtatttttgtataaatgagtcataaaacaatggttaaaggtcgtaaatgttgcgcgggcgcttcgattcggttggtcctcttcggtttcactcggccgtcggtcgtcggcgctcggaccgcccctacccgcagacggcaatgggcgggactttacttatacattttacatagaacattttatacttaaaactaatacctaataactactaatagcctataattactgtttgggtaatcaaattaatgtgaattgattaataaacagtacattaacaatttaaataaaaaatctgaatagtttatatatcacaataaacgttgtttttaatttaaaataggttagtagttacataagcaaattaaattatcgaaataccaatacaatgaattccttagaacgttttagcgtttatcgtcttatttaaagtttaaagtaattaaaacggttgtacctacattaactaattatcattaacaatatattcaagtaattacatattcttaaaagctaatctagtaggaaaatggctattaaaacggaatgtattttgaaagagaatgcattaaaatattttactgttggctaatttaatttaagtatgttttagttgaaatatttgtaagaagacacttataataaaacactacaaaggaaataggtcaaaaatataaagttactaaatatatacatatattactactacatactttatatacttatatataactttatatatatttctaaagtttattaaaatatacgtacagactttaacattatatatttgctcgtatagagtaccaaaaccatttaatcttgtgaggtctacttagtactcaaaaaaggcgaacgattacagtctcgaagtgcctgtacgctttgcatacaatagcgcaattatttttattatactcctttttattagcttaccatcaatcttccgttattatagggctaaatatcgcattcaattggattggatgattagaaaaacgttttaaatagtttttatgggacagaattaggaaacggattcacgaatttatattttcagtttatatatctatagtaaataaagtttaaattatataatatatggtaataggtatacaagtaggtatagtaaaaattattattatataaagacattttggaataaaatagttaattattttcttaagtatatatttcaaaatatacctagataaaaatttattaataggaatttattttaattttataccttttatttataaaaaaatcacatatttaggtatatatattatatatttttattgtgcagattaccttcttgtacataacatataattaattgagagttagatattaattaatgattatccagtatttgggaggcagatacttttataataaactctcaattaaaagtaggtaaatatataggtacgtataaatgggcttgacagtggttgattgatttatcatacttgtattacgtattaaattaagtaaatactatttataaaagtaaaattataccttaaaataattaaattcatttccattctttttaccacccaattaaatctaaattgcagagtttgaaaataaacaatgaaaaagaagaataatattaaataaaaaaatatcttaaatgtactaatgtactttatttatttataaacataccaacaattacttactttaatttataaagataatttaaatctgttgagatgtaaattttactataataatagacaaattataaataattctgaatttaaaaaataatttgaattagaatggtcctatttatatacatataacaacataaaacaatttttattttatctatagacgcaatggcaataataaaatatatctacaagtaaattttgaaataagtagatacctaagcataaaaataacacattaggtaattaaaacttattttaacgcaatattaattaatataaaaataagaaaccaattttatgtgacaagacaaagttgtataaatcaatatattacctatttgaataccaattataaattaaacataatataattaaatgtatataatttttatctcaccattttaaatttagttaatatctttccgatattttatataaagtataaatgttaatttatggatgatttcatcggtaatatttattataataaccttcgtacataagacgataaatcaatcaattaagactattatattgtaactttagtataccaaagcttatatattggtacctataccataaaaacaaaattaaaaaaaacatattaggtacctattttacttgaatgtttcgttgcctttaattatgttacttaaaataaataattaaaaataaaaatcctagtaaaatcaatcttaccatagaaaatcaaataggctagatagtatttttaacacttacaagactaaaaatcataattgaattaaaataatatgaatggatccataaaaaatattagaaattcaaaacggaagtctagagcaccgtttataaaattccatttcactgccgattccaaaatacctaagcaattatagaactctcaaaaaagcgtaatcgcaagacttaatttgataaactacatcataattatgccaaaaaaattctgcgtataaatatcttagattgcggtgtagttatagttagacttccataagatatgtgtgaaacaaactttttttctacctaacactgaatcgacgctcttggcacagacggcccagtgtcatgcaaaaaaaatcaaagccaatgaaacgtcataacagcctcatacatcattggaattatataaaataggctataatcatcttgtttttgtttagcaatggactatttttgtgatttgtcggcaatgttggcatataaaaatgtccgggctgatgtaggtgaaaattcgtctcgggaagataaaaatatcgcggcaggtgcttgcgcgcgcgcacacgaaattaaaatggcggacttgtcagtagccgacagttatacgtcgtaacgcgcaacattctacaacaatttgacagtataagagcgtattacgttcaaacactacactagtgttagtttgaatgaactttagtgcgattcaatgtttcttgtttttcatcgtatactgttgaatggaaaaaactcaacacgtgtctcagatttatacatccaatgtttccccaaacattcaattataagatttaactataataaacaaaagggtgcgtaagaataaataagacacccataggtagtttaattcggtaatattttaagtgaaatcaaaacatttcttgctagatacttcaataacaataacaaagcagtttttattaaaacacctagtcattttaacatcaaacccaacgcgcatttgacagtaaacatggtgtaccgcggaagagaagaaccccattcacatgtgtgaagcgtgtaaaagcgcatgcgccaaacaaatatggccgtcgtttacagccaatcgtagcgcggcgtgagcaatcggaacccacatgccatcaaaagccctttatactattgattgctatttgtaacacgttattaattaaattgtgatagtcatttcaaacatttaagtattttaggcacatgttgctactaaacaaagtaaggtagatacaatttacttaggtacattaaattaaagatttaaagaatcttgattcattcgcacatgtttacgaccttctactctgttgcagggtttccaaaaacaataaaaacatttaagttgaaaaataacatcattatcttttcccagtgaaatatgggactgatacaaaacttttaaccttttccattttagggttgcaccggctttcgaaagtttcgacagtttaaaattaaaaaaccgaaagcctgttgttatctcgaaatgagttgtgaatttttgagtatcgatatacgacgtacgattcgtataatcgattttttatgcattcgccgtgtaattaacgactgcactcaatcgagacaatttaaatgtttccgatagatacctataactcgttaattttaaacgttgcttgaactgctactcgctcaatatttttgattggacattcagttttagttcttatatttgcagagataatattttaaaacatcattacctcatttttctctctaagccgattttatttagctctctgttcaaatataactttttgattttgatgtaatgccagaaaattttaataggataataattcaataacagatgtcatgtctttataaatgataactgctttacgtagagttttacgtttattctgagcctacgtatatattttttgcttctaattaataacccttaaataacctacacattgtggcctacgcaaaaataataatattcgattttaaacacagtgcaattttggtctccgttaaatacgttttttctcaaatatatttgtttttaaaaacaaaactatattagttagaaataaggagttataacacattattatccaatgtactagtgtttaatctttagacaaaatctagtgttattttgaactctcttcacacgcacgtgtgagttggtttttatttgtaaaagaaatcaactgtagctatgattcattgtacaagattggatcaaatttactgtaaatatctatacatagttggcaatttatttagctcccagactacggtgctatttgaattaacattgttgtactcagcgctcaataaaaacgaaataacagccttaattgataaagtgtaattaaaaatgttttactcgtaatttagattcgcgattaaaagtaattaacagtcaattcttctagtagtttatactcgtttaaaataggtaagcattgtctttgtggaaatgcttaatttcatttttacggtcgattttatctttcaatagtagtattgttgtaataaaaactattataggtttttaggaatttttaatctattgttagatactaaaactattatagtaccagcccacattgattttagaataaaattcatattcacatttttaaatcctattctattcctatatttttatgtatttgcaaaaacaagtgtaaatagtataataggtgaggcttgttggattgcgcttattttgaaggattagaaaaccgctgtaccatttattctattaccttttaacagttatacaagtaggaagtatattttactttaaattacctacccaatgcggtgttcttatttatttttacctcaccattcaatagtatgaacgttttactttttatttattgtttaggctttaagtgttgtgcatgtgtcttggccgaaactatgaactttcaatacgattgataata***GATTTTCCATTTCCAGATTGGATGAGCCCCTTCGACCGGGTGGTCTGCGGTGAGTTCAATGG***tatttatcgtttcaaatttattttcttttagttttaagtatatttagtttaggcgtaggaatcttaacacacatacgactaaacagcattgttaatggttactaatacgcttcggaattctcaatctgaagtgatattttaaaacaataatgacagaacttttaaaaaccacttcttctgacatttttgatataaaaacgtgaaataatgtcagataaattgttttgaattgaataaagtcgtttcgtattccatttgggctgagaaatgactgtgtcaaaaatattattttgaggcctttcaatggcagtttttgttcaaaactaggtcaggtgatagacaaaatataattgtcttcagtcacaacattggttgcttatactgctgactagaattcattgttttaaagggtttcaaagaaaaataaaacataacagtaaagggataaaactgtgatttctttgtcgaatgtgtgtgatgattcaagttaaaaaatcgtttgaaggctgtcgtaaaattatgttgcaattattaatttgatatggctttgtggaattattgcaatcaaacctttttttgacaattaattatttttacaatattattttaatttatatttaaggttttttaaattttattggacgatttcttttctgtgtacctattttgtgtcggtgaacaacacctacaaagaatttgttttttttttaaatcatcaaaggtgtattgataaacccttcaatgctacctgtattttgcatcacgtatcacggttgtttacgcgtctctttttgcgaaaaaatctatctttcattgtttcttttttgtcaccatcgagaatggacactcgcaaatctattgtcctgttgcaaaaaagtgacgaaaaagggtttaaaaggttcgcaataaatacaaactttctctaagaacaaaaggttttaatagtgggtcaacattacgcaaccaaacattaaggtacaaaaatggtcaaggactttcaatttttggtaactggtgttccgtatagataagctccacaattatttcccttggccgttttcctttcgtatttacaacgcgggcgtattttcctcccaaaagtaatataaataagcaagttgggagcacccgagtagcatttaaatcccccatctttaataactataacaataaatttttagttgatggccgcatcccggtccttcaaacgcaatcttgaaacggaacgtgttttgtaaacgatttaacatttttgccttcctatatagtagttatgtttttataggtatctatttgctgtgtacctacttatttattattattatatcggcggtaatcgattttatatcgatatataggtacacggttttagttactttgaaatagctcacggaaccataaaactcgagcgtagagacaagttactttcacaaatattttttgtacatttattgaaaaatctaaacaaaaggtacgttgcaatagggttgcgcaaatttctctttttttattataaacaacaatccctttgtgctcacaagatgtttacaaataaggtataagtcaaagggttttaaatcacggcgtcgaaagcatttaaaacaagcttagaaagggttccattgttttgttggcacgcccgcaaattgctttattacggacgctttgcggctgttgaaagtcgatatcgccaaaatcgtagtcaaaacattatttgttatcatacggtgttctgcgccacaagtcaggacagaacccgcacttataataaacgtaaccacgacgctcacgtgccttcacattgcatacctacatgcgagtcctctatatccatcttcaaatataaatctatcacttcgccagctcgtttattcaattgtgaaagtacgttctcaacacaattttatcaccagatgacgttattaatatacaaacagtgtatcaatgtgaaaaatggataatcataacagttacagagtatataaaaaggagttttatagcgtcataacaattaagacttaatcactgtcaatcgtaactctaccgttataaagtgattgacgctagtgttgtaaaaaaaagtttgtccgcgaacaataactgttgagaattgaccataaagtggcgaccttgtggcaataaaaatttaacgagccaagcccgtggcgtagtgtagtacaaacacattgtcagattcgtgaacgtgactgtcatagaaacagctgggcccacatctgtgaaccgctccagcaacagactcagtctttttaaaagtcattaaaaagtaaagagttgggaaacaacgattgatttattcacgggcgtttaaaatctttccgcgtaaactcgtttggtaaaaccaatcgaagatttattgttttattttttattttatttgttatacaatcgactatcgctgtgttgtcgatgttttaacgtctcaacaccagtataataacttcgtctgtagtaattcgatgcgatcataatatacgagtgttttaatacgagctgcggacgtttatgagttgtaggcgccaaagtattttagacggaaattaatttccataatggtctatttgaccaccaagtcgcgttgtcatgttgcgcattgcgaatgcgtagatgcacgagcacgactccgtcgcgtcgccaaataagttacctttgtacatgcataaaatggtagataccgatgccttattctgctatctcttggctcgctctactgtgttgatatttcaagcatccattttaacattaagatgtcttattttgaacttattctctgcacggaaagatagagggcgcaagttattaagcaatgcgagagatataatgttttcttgttttctttacttattcgtcgtttataaactacttggtcgacataaatcagctttcttaaggtcaacaggttttataattaagaacagtgaataagtaggtactttaatataatcttatcgttattccgaacgatctgtttgtgcatagtttcaatgtctgcgaaaactttattaaaataatatacctagtgataattttaaattaaactaaaacctaatttaagtgcggtcataaattccgtaaaagggaacgaatatgaaagagttaaaacattttatcaaagtagaaacatgtgttggcctacaacattttttattcaagttcgttcaatgaataatgtcaatgccgttcttgtatcttacaacacaacatggtctgcgttgtactctgattactcacactatcaaatgtgtattcattagacgaacaagacaattgtgcgctacccaaggttgtatttgttgatgctgccacattgcgcaacgttactatatttggccaagaaaactaaaagttcatcgcccctgaaaattgtagtacaactgacaaatctctgatttactaaacacgtttcgctttaaccaatttcagtataaatacaaatttacctaattatattaatttatttgctcaataatttagttcttagactgtattaatattcattttcgtttctcaattagctagaaaggataagctgtatttcaagcttgagtaatgaatatttattctgtggattgtcaaaattgtattaatttattggtcggtagattagatccgtaggaagattaataattatgagtattaagtcatacttaggtactaagtctaataggcttaggacttgtacactaaatgtaaacaaccataattacaacattttaactgagtaatttagatctattcgtagttttaaccagtatttatgctaaaataattaattaagtattaattaaaatttagatttgagtccgcatatatatgaatatgaatattcaaacctcaataaataggaccaatttatagtatatcaacctaatatagctgtttacaagcataatatttcacatgaactgacaggcttacaaagtatatacaaaaatgttcgaattaacgtcataatgtagggttatttataatgaaacttcaaacgcatgcaaaattacattactcaagaaaaccattagtgtagattatttgattaacatgactttaaataatacatgcccaagcagcactaagatcttaaacagaccagttctacatgaaatagtaaaaccatcaataccattatacacttcatcactacacattaaaaaaacaaatttccatcgtctatctgtcgatatgtatcttcaggataatctcgaaaattactcgcctgattttggtacgatttctatcgccacttcaactgttttctaggaagattttgatatattgactacgcgtgctagtaagatatagataggagccaaactgaatatgaaaacaaacaaacaacagatccattttttatttaaacttcatgaatgaaacgggttctttaattaaattatacaaacattgctgaatgtacacaggcctcatatttcaacaaattcacggaatcagattcagctaatgattttaataaaacagtaaaacggaccatgcgcgacgtccaagttttggaactcaacacatttcttggaatattttattttgtgaataagccgtaagtacatgacagaggttgtctccgtcttgaattgttggactcaatataccacaattaacattctgactccattcttaaccttgttaattcttagaacgtaaattatacctcagtcaaattacattgcaaagaaatattcagccatcttgacttcttcctttcatttcagtaggtattaataataatgattgccctttatatacttggaatgtaaaatttcaatagattcctttgtgataaggatggcataaaaattcaacgaaaaagtgtttatttttatacgataaggtgcatttaaaggtgttatttgcaattttaatttgcatcctgttggcataaaactaatattttattacaaacctgactatcgatcaagttgtttgaattcactaaataatacatttcacactaccactacttgtgatgcattcaaatacaactcttatagtgtactaacactgaattatattttactgaaatcgtgtacaacttttttattttatatttagaactttatagattgtgctgtataattttataataaagttctttataacattttgaatttttggtatgtatcaaaaaactagaaactactctcttattttctttcgtgtgtgtttttaatgaaaatccttagttttatatacgaattcaacttgctatttaaaaaataactatggtgttgtttcaccatcatcagtctttgagagactactgatggggtttagaagtttgtcataatctctggcaaagcgggcgtagatcaaagtatagtcacatagcactgatttgttaagcacacaaaacgtttcatgcaaacagaagtttgacattcaaataggtattattaatttgtttattcgatctaaaatttaattaattgcaaattgcatttaattaacatttataaaattaacgaaagaataaataaatgcaggtgactttgagttttactaatttcttgtttgctgtatagaatagatttattagcacacatataacatattcgaataggtatcgaaaaatgattacatcgatcaaactcttaaacaaaaagtgtttaaattcttagaatctgggaaggatggaagccagtagtgaggtaaaaaaactttagatgagcattataaatggcgtagaactatgtaggtaggtacctattggaaatccacataaatatttaacagtcaacacgtttacctggaactcaaaataaataaatataataaatgtttaatgcattactttactgaattaatgatacgtgtatactgtatgactgatttatacagcaggagatcagataacgtattccatgttgatttatatatttataaagcagctaattttcattccaaattattgactgaaacaacaatgttctgcgcagtaatacatagggttatgtacgtaatacagcgtgttattagtacataacgctcacaaacgtgactcaatagcttcaagaatgtgaacggaggcattgttcaacagtccgtcgaactttctgaaagctttagtttgcttcgtttatgccaactacgttatgctaatatagatacgatgtcgtctgctagttcactactacgaaatttattttattcgtctgctatgacttgaaatttagctaccagctaatgctaatgttacattgcagacagtttattggttatttcaaaattgacgctgtcattacttagaacgtatggtgctacaaataaatatattggtcgctttatgctcgttacaatgaatgcgatgttggtttaggatccgctagcgccccggagagtttgatctaacgtttgttacacatagctgcggcctaaagttagaaagtccacatgaaggcgacgaaacaacgtggcaacgcgtctgttccacccgataacggagtttcgtaacgctataggagctgattgctttcaaagggattaaatggaatgcgtacaatttatatctctattaacgtagagtattgtttacacacgcctgaccgcggcctcggaggccaaacgcagacgcttgtgaaaacattcgcttcgagattaaaggtagacctggccaccaatgccgcttcgttctgacccgtttagtgccgcccaatcagtaatgcgtcgtgctccctcagaggtcaacatatattagtcgcgtttaagtttgtttacagcgggctcagcgtttccgaccagtttgcataactcgagggtagtctcaataaaaacaaattggctcgctgaccagagttgcgcattacacacgtacgaggaagccgggcgacatgcccggaggtgatttactgacatttcatgccactaagctcgaacggttatggaaaatagatgtaaactacgattatgttctaagctccgaaacgttactctttatgcgatctcgaaagtttacatgagcgtatacaatatacaatgtgtgaaaaacatttaaagcatataatataataagaacaagatttaatagataggtactacggtatctacaaagcctacccaagcaaatagaaccagccattaattttcatacttaatcccatgtaatgctaaaaacattattcacaatctaaacgcataaataagactaagctatgtataatttcaacattcgcagatacataattgaattaatgttaatgggtataaccatgccagttatcagctaacatcaaaaatcctatggtaaacaagtcgtattaaatcagtgggaaggtagtacataaggcgaagaaaggaacatcggctttacctacctataggccgttagggctgttcgtacgacactcttgcgcattaatctcagagatacaatcgcggtcacagcgagctccgtaaatctcagatacggatcttgatatatggtggggagagtgggggtatgtttcacctttgtaaggccttttgcggaatggggcgacacctcgagcccgatacgggttcggcattgttcggatcgtatcgtgtacgctcgttttgtttctttatgttacggacagcggtcgatgggcaatggagtcgaaacgatcttaaaatttttaggcgattgttagatctttgaatgagtttatttgcctagttatattatgaatatatgttagaaccattgaattcatttatgtaaaagttccaacattctttatagtcaatagacaaacatatttaacataaaatacgtcagtttcacgcataacttttacggagagataagtaaagactataaagcttttttcctttctagttcctcgtctcatcctacgaaagagtctaataaaaattaatgtgaacattcttacagatttatgacaaaagttgagtaaaatcaaacagatgctttgatctgcgtgttaaaatgttatgaaatcatataggacaacttttgtaacatttacacaccgtcaaacagtctgtgtgctcgttacacataacaaatgacgtcattattaaagagattttgtgtgacccacaaatttacattcccgctcccaaaacacaaaagggaatacctaaggaattttttttgttggttttttaaacaagaaaaatcacttcgcttcatcctgtcctggtggaaggtaaaaggcatcattggcccgctccaaccaattatggcgacgttcagagtaatttaatcgccactaataagaaattaaaacttgattttattgtagagaagcttaattgtacgatatggagatttttgacgcgcctgccttttgaggtcagccgactcctttaatgcgatactcgaattaataacgaaaatatattaatgactttatctaggtatatggtaaatggtatttgcaatgtcattctcatcttacattcgtgtaacgaacatgaattatggatttaatctaggccccgctcgttttgcctacggtttatgtaaatgaggcttggaaaattgtaatcaagaattgttaattacaagcggttaccacccacgacttgtttttgcttattcgtaattttattatgaacataaaggaaattttatttagtccggatatacttatatagtacttagcctttctactacaatttacttattagatgtaacacataagtttatgttgatacgtatcataagtttccggaaaactaaacttagatgctttaagagataactttgcctttgtttttttcttatacagacgaattttgtttgttcagtctaatgttgggttgagatcaacaaaacgcttaaagtacctaacagagcacatcattcatattgcgtaaactgctagaatttacttgcgtcgatattacttcaatgtatactgaaaagtagaaaaaaatctatttcatgtattagtatagcaatacataagtacttaaacgcacaacgtattttcatcaagagcagtcaaacatgatacgagtgttgcagctcaatcgctcgatataaatagtgatgcttacatctgtaatcttaaaatacgccgtgtttgataatcgataatgtaaacgattttatacatcaatgaagcaaacacacatatgagtggccagccctttaacgtaattaaggcaatatttctatatatttttgtaataataccagttatttagcatgtttcttagtattcaccgaccggatactgagtcgcgataaattccatgtttacatagcgatgtgtatctccgttatggacgacaagatttcaatgagtcgcgagttgtgctactactttgtagttggggaagtaaccgaggggagaagtacctaacccttccactgcacatgatgtacgaggtgccgaatttgaaattataatttgacaataaaatccatgcaattgatataattttatacagccgttagcattcgagtggtgttgtgtaatgtatgttggagggataggaagggccatccgtggggtttaggggatcaggggacaggcgcagggtgcgtctgacattgaagaagtgccaataacagaggtataaatagcgggggccggcggctgagggcgttacttcgctacgagatcagaatattttgttagttcgctccgcatactgtcctaaacaacggattacaatgcgggaaggcgactactgaacgataccgatctatatctacttgataaattgtaaagctgatacaaatttatgggttttacgtgtcgcttgacacttaatgtaaaacatttatgtatgagtacttaacatttaaactggtagtatgtaggcacaataatcgtttcacacgaaggtaacttgtttaacctcggacaatgatacctaagtaattttgtttaacaaatccttttacaaaaaggtttttgtcagagatttcaaagacttcgtcacattgtaaatcaaattgttaactgattaactttggtacgtgatccgtaaaggtagtccatttgaacgattcaataacttggagttcacaatgagtgaaacaggtttcggtaaacattgttagttcgttacttcgttagtaagctgaccacacgactggtcctaaggtggtattagcccgaaaaaactacaataaacctcataaattctcccctaaaaattggtcaccctcgaaatagacaaaaatgaccagaataaattggaatggtagtggcagccgataaactattcagtttatggggtgtcatatttacattcgggaagatatcgacgtgggaatacagataagtacttattttggtggacgtggtgaacacaatatgttcctattcaactccatgtatttatttaagtgatataaatctacgaacacaacacatggaacaaattacgcttggggataaaaagaccacattacgtagatacctacgcactggctgtttccgagtgaaatgtttgtgtatatacatttaccttttggttcattacttaaggaaataataggggctttccattgagtccgttaatccaatcacggtagcactccaattaaacaaagccctctggctggcgctaatcgattgccaataaattgggattcgagcggactctgaacgcataatttcttgcaccctgcggttgtgtaatgtgatgttgcctgggggtgcaaactcatgtaaatctatggattatgtgcgtaccctagctgccgaggtgcagataatatgcaaacggggattacggatgggtagtacccactaatattattaatagcaaataggatgcaaaatacagtaatatgccacattttgaagcagatccgtatttcattccctgtaagtagcaaaaatgagctttatatacgcaaggtgctccaacaaaaaataaaacgtattattttatgttacgcgctctcgttaaagccccatacgaacgcgtgtaatctccagacatatttatttgaaaaacgcgcacaaacacccctaaatggtagctcaagattagtcggagccattccaacacgttacgtgaatagcgatgctaccaaataaaatgtttagggtcattttggccgacaacgagcgcgcctagctcacccaaatgccaataggtctattttacccccacggcctgaggggagctatacggccatcataaattgtccgccgcaaatttgcaatcctatcttttttggcccggtgagggaggtccgacattaacataactgtatgttgggcaattctacgtgtagctttatgaacataacagctcacccaagctcgtatatatgcttgaaacgtcatgttaaagaccttatatcacggtcccgtaatgtaagtaaaggtgcgtcttaaataactcccagaattactctatcattctgcatattttgtgtagatatatgtaatgaaaactgttttgaactgaagggtagcatttcaaagacgtaagtaataaatgtaagcaacgagtttcttcataacattaccatccatattaatgcctcgatttaaaattctaaaaattacaaagaagaaatttaatgaaacttcatgaatctggattaatttttaattataattgcggcattgcaaatggcggactccgtccctggaacgcgtgagaggcgcgatttaatcacttgtcttataacaagggtgggggcttggggggaaatattaagcgcgttcttgagttattaaagccccttaattgcgcttagatattttctggttcattaaatacttaagatttataacgagacacttgaatggacgagataagtagcgtgacggaaatgagcctatcttacggtgtgattaatatcaactttgggaatttgtcgtgttatctacattttctatcgagacaagtgtaagaaaaaactgggccgtgggttgctttatagacgcaccgggaaaacgatatttctcactccaatgtcgtgaaacatccggcctattaaaaacttatgacacaaaactattttatacgatgaactacgtataaacagctaaattatagcctattataattaactgggtaaacactgtgactgtttaacacagtaatacaaacacgaaactgggtcctttaagcgacaacacgcttggaacgtggccctatcatagtttacaagcacataagggagcctgcgtcgttgggcgcttaagggcgcattacacccttggaatagcagctccgtaattaaaacgctttaaactgactattacgaagtacctatgaaaataacacgccggcctcctatgagcccttcaagagcttagatgctggcaaaattgctatgaaatttcatagcagagaaaccgcattgcaatagaaacccatagacatttcagtcctcattcacggctatcaattgttcattagtttgcaaattcgagctgaaacgtcttttatgcataatggtattttcattcaaaatcttaacgattttacgtatcgacgtagtcttgaactcactgcgttcgtgccagaaggttttcaactctacgtcttaaacataaagctttaaatgatttgtaatcttttaaacaagatcgctattctttgtttgtctagattgcggagatcatagtcagttagtctaatattatatttttatgtactgtttgatctcttacattacatacagattatgaatttattccgagaattattttagcgtttcctcgacacgagaaatatgtgggtacattcaactttgtttactacaaaatatatagtgtagcttatctctaatttgaagtagcagatatccagcgttgaggattatatctccactatatctttgcccgcattaactttcaattaaaacaatttatgcacccaacgcacagcgttccagcgttgaaaattgtatcgtcataaattatacagttgaattaataagtgtacaatagacgagctaacgagctcggtcggcggcgaccgcctgggtgcatacgagcaaattaaatttgcatgtttgcaaccccataacaccccttcgctccgccctgtccgtccgtacgggagttattagcagcagccgccatttttggccagactgaccgtagggtgaaccactgggggataatgtggtcggcaagcaatttgttttcggatttagttttgtaattggaagcgtttcacactcacgcagccgtacgttagctaccaaagtaccgtgaaataggcgcagagatgttacgacacttccccaaacttacgagttagcactaagtcgataacacagacgagcccattactgatcaactagctactcaagacctaagcagatagaataatgttcttttctcccaaagagtgaagctattaaatagtcgtattccgcaacactatcagacgaaacaacaatttctccggagataaaagcagccatttttgtgcgaaagctctctctacctagacattcagtacattaggggggtctattctaaaaaacatttgcgcttctcgcaaggggtgggcgggtttgaaattataaaatataacaacattgcgccctttgttttttaaaggtatcgtacctatgtgatggggcgcgagcatccgcgcagtattcaaatttgggagccccgcaggggtaactaattcgttttaaattacgttacgatattgcgtttcgacttaaaatatagctgatataaacaagcaggagagggttcgaataaaatattaggaggcactcaggttcgcgctgtaattagattacctattagatacgcacacgtatcgcaatccgagccggattgtgatatctgtgttgtgatgctgaattctttataatattcacatggacgtttatcaattattttatcaagattaatgaaataaataagtaaacattgttatatacttgggaaaaggtcacatagatagatcacaatatttattagtgattggctaaaatatttcgtgatgagacctgcgttcatcgacaacataatatagttacctatctaaaattttccaatagtgtttaaactaaggaaatgccagacaatcaatattaagtactggaaacaaatacacacaaaatatgcagattgcacaactttgtttaacttctaacccacatttgcgtaaacataaaaactaaaactgactcattttgcagttcggtctttaaaatccaagttttccacccactgagataatttataactgtaagagtgcaaccctgttatcgcaacatgttcgcacgatatttattagacaggtgcatattttattagctttcgaacataaaacaacacataaaaactatcactctctgacgctcaatgaacaagcccggaacacaaatcttattcgcaataaacgagacgaaacgaaacgcgaacgttaataaagatataataataaatattcggaggaaaacctttcgggattcgaattaaactaagggtaggaaaattgcgagagtccccaaggaggtgggcgagcgacagctgcgagcctaatgtcctttctatttatctgtgatcggggaaaagctgtatagatatatagtacatcataacagtaaagtatatgatattcccgatacgacgcatcgcgcgacagatgtgccgatataacaaacacacgattaataacataatcgacgcccttctgcatgactttcgaagacacgtctcttagaaatcgtgccaatttcaacgaactccactaaatattcattaaaacatatccggtgtttatgatttaatgccaataaaaactattcagcaaacaaaacgaattaatacaacatgatcgcctattcgcactgatattgcgtaagtcgtgatgacaatctctgttttatggtgtagacctcgtaaatgtctattaatatgaatgggctccaattacttttacgattcgcgttttgtcgagagacgttctaaaaaactttacgatcttccgcgcccctggtctgtttgagtttacgtctagctgagggtttgaggccattcaggactgattgaagcgtggacaatcggttcgttggcagatgttgcacggaaagttgtcacctgtccaaaactgccttgactctgacactcggacgaataagcaaacccatttataggttctgtcgccaatcgaacaatttgctcctagtttggtgtaagcagtttgtgaacaatgtctataattgagaaaacttcaaaggtatacttggtaattttagaccatcgcaacacaccacaccatactcaccaaataaatggacggatatcagatgatattcagaacgtatgaatggtacatttatatttgtatctcaaacgcgacgatgaagcaattacgatgcaacatgcgcctggtgaagtggtgaacgattttattacaatttaaatttcatgtttatttactccactgaattattttatatgctcagtaatttgcttttaatgttcagcttctttatttagtatagccctttgtatgtcgagccaaatatgttagcccttcaggtagaaaatttatcgcatataaaacatttacacatgagtcatagtaatttggttgatatgtccagtagaagcgtcagatgtattagcatacaaagacatgaaacaaacaaaatcggtagcagaacatctgaaatgaacaataattcccattcagtgaatcagaatgcgttgaagtgaatgactatggaccaataatgtaatgccaacttcctctagtgtgtgccagcgtgccgatgccggtcagggcgtctatcacatcatatgacccgctgaatgcgtcttattacacttttatcggtcgtatcagtcataaaatgctaatgaacgagcgtttcggaccaaacatccgagcgagagattcaaatgacggtataaaacgtaacccgaagtcgacacttcgttggaaacgtaatcgtaatcggtctatgcgctttcaaaacaacgcgtaacacgagcgtctgtatggccgcagagataccacacgatgtttttaaaagctcttttattgtgcgaccgatatacctaatacaaaaatgtacactgactctttgcttttgtattctgtgctcaatagcttaagacgtagaaacgaagtttgctcggaaaaatgtcgctacagaaaacggaggctacctacgtagaccgtcatcgtgattcataacttctttcggatttttacaacgtacaatggcatttaagtgctaatatttcttaatatatgcgacgataatatttttaattacaataaagcactgttgtaaaactgtaattggttataacacatttgtttattgtacgtgactacgtcattttgttttccttattgctcccgtataaaagttctattcatgttatttttatgttaatacttctcattttctttttgtgtttagcttgtaaattgtaagattgagtttaacgcttttgggtctgttttaaaataaaatgttaaaataaatcagtataccaacttccaattattctattttatgaaagcaatgataatatagatgtcaaataaaaaagatctattaaaatgagaaataaaatgtataaacgagaggcgttgacttatttatttagtgtaatgttataaaactgtattattatatacttgaagatggttccttagtgctatgtttttgtgggaataaatcaattaagtcactcgtccatttaaataacaaaaacgtcacagaaatatccaacggagagaactgaacaatatttagttacctcaagattgtacgtgacacttaatactgagcattacagctttttttgtgacgcccctggaacgttatctgtgactgatggagccattttctaaaataaaacggccctctcctcttcctatgagaatatacgaccacttcacattaatatattatcaaaataacgctttctgagctacgagtgtgcttacgagcaagcggcttatctacaaacacgaatactcacaaataatatctaataggaaatgaagtatgtacttagcgaagcaatttggagtttcaaacatcctgttatagtgttaacacgtgggctcctaatataaataatgatgacgaaaaaattaaagcagtgtaagtcaattgtttccgggagttatatttcaaacattcgggggtttggatcaaacaaaacaattcttttgaacatgttaactatttgtgtgtaggtacagtaacagtgatacgttcgcctgagtcagtggtggaagaatgcaaaaagttcttaattaaaacatcagaggcaaaaggaattaatcccttcattgcggtgggcggtagactgcacctattttatttgtgtcactaacgtatatttcagacgtagcttaaaagggggaaatccttactgaaaattccctattagtttttactgatccaaaatgatccctatggcctcaaagataaggactgaaatcgttggtgtgtcggttggagtttcgacttttgtcaggtgggttgagtgccccgtgcagactttgtataattacgttctgtgggatttggcgtttggaggcaatgtaattataacttgcactcgtatgcaccaacaacggggtgttttttgtctacagtgaagcatcgctggagtaattagcgagcggctttaattatttccattaacattaatataaaattcttatatttttcattatagaaatataaaaagcgtaggtagcctcgtatatctgccaaaaaacacactaccaattttccagtgcaaattaaaaataaataataaaccacaagattttcgctaacgaaccaccggacgccataaaaacacttcaacatcgtttgacacaaacagctgctccactagtcaactgaaaaaaaacttaaacactaaaaattgtgagtcacaaaaattaaatgaaagaaaataatacaaaaatcgtgcgcgcgagtgttcctcagaagtgttcttcattggccattctgcaaacgttacgaatgctccttcacatacaaactggtttggttgagctactgagaatacatgcgttgacaatgatctatgtctctgagataactttctgtcaaaatgcgcttgcgctcccctcgctcttaaataggaagcttgttaactgtaactggctgatacattgtgtacagtcgaggaaaaaatacgtctgcagctacatgcaacgacttttaaaactgtatttaaaatgcagtaattgatcggaaaatattaaacatagtttgtctgatcttttgacatttacaaaaattgcattattttaaattttagtcgcgtttttgtctgtcttgtctgttctgcgcataaaacttttaatgtgactgtacttcattccaagagattttaatctagactgtaaatcttgcctagtctactagtagtattattcagtaggtcagtgatgtttctatttaataatggtaactaacttttcaaggacggtcatattacgtcacacatggtttacattttttaaatatgtttttaaagacaattctaattctttttttgaatatatctatcgtttcatgatctcttatatttttttgtatcattatgttcctaaatgtatttctattttcggaagacttgacaataaatcgagatgtgaacagctgtcgcagtgtttaccctttaatccctgccttatagttatatattattctcacagttagtttacttataagaaaaatattatcattagaataaacataaatatgcgtgaatacgcgtctttgcttttttgttgaacagatgtccacgacatattttatgtaggtactattttttctattcattcaatcagccagtggctttaaagacggtagttggttaaatttacaatatgaatcgagtcacgttgatccctaatactttcaactatggtcaatttatctataccaatatattttctacctttaatataacataaatgattgaaaacataactgtcaaacctcaatcgaatatataacagtaaatggccactaaatactggttttatgatagaaatttatctaataagagataaaattttatgtaatcgtcgctaaatcgatatctggactattacttctgtttaagtttcctataaacgtttttcttaagaagtagatgtagttttatatccgtgattaacaagttcgtttgtttaagaactattttcgcgtataatcgcgtaggcattatttcgtagcatcgcctacgacatccgtagtattggcgtagcaggtgttatttgaccacgaaattaacatttttatgaaggtggcattaaaattagctacgttattattttatccaatattttgatatataactcaattatcatattataggtagatattaaaataggttgctacttatattacgttaaaatcaatgcgcagaaattgatttatttccgcatgtcgactatcaatcaaaggagataagaaaatattttatatcactctgtttctaagcaacgaccactctgtatatcaaggttggagtacctattaacgtattgagatatgcatacataaataccaaagtatgtacttataagaccttcagtcatacctaagaaccgccacagaatacaaacagctgatagagtaaacgtttcctttaattccttgatcaaccgcgtagctactactatggatacaaattacgtataggtgactcgataatggaaacattcagactaattaaatcgaatattatatgtcgattgcgataccatttgacccatttatctcgaataaagtcattcatttcaataatagctgtcataatattcacaatgtatgagtggccatagtaatgcgttttatatacttagatatattatatgtataaagaatctgcaaaactgtatctactgttcattattatgtataattgagtcattgacgatgattatagaagtttttattgtttatttacaaagtcgatgttttattggtatttaacaacttagtagcggtgctaaagtaattagatataatatatgagctaggttttatgtgttttatacagtttgtatataatgagaggaaactgcaaggaaaattattttaattgttcttcaccgacaaaggctataaataaagttgttttaattaactcttacgttataacgtttgctaacttaattggcacgtaaggtacgtattaagttagcgattttttaagttaagaattttagtgtaatatcggtcccaattatatacacgttcgattacaaactcacgcaaagctttatacaactctttgtctcttaaattctcagttgtatctacatttcatctctagactaacactttattacaattacaagaaatctgtagtaactgagaaaaatctgtattacatcaaatatacctttacctatcccgtcataggataaaaggcatgaggctattgtatacgtaaacgttaagtaatttatagatttagaaatagcgcgcatgcgcctaacgtaggacgccatgtcatataacacaacacttgtaaatcgttcttcacgcattttaacgtgtttcctaactcagtctatttacagcagttactgatacatttaactggtataattttatcttttatcatttaaattgtattgcaaaagagttaatccataatttttagtttatttcgaagttaagatctgtgaacattattaagaattaaccaagttaaggataatagagtttgaaatatatcatcattatacaagtatagtattaacaccgaaccatacagctatgtttttaaataaacacttgcccaaaaatacctttctcaaacaaaaatcaaaaacaaatcagaatcaaagctttctgaaaattatctaccacctacataaattccaaaaccataaaacagcattcaagaatttatacggaagtttaacaacagaaatttctagcatttaatttagaaaattaacatgtaaaataattatatggacacagtttagttattttgaaaaggaacgtaattgtactatggtagaataacagctaattatcacctgcttattatgcaaattaacagttaaagtagtaatttctattaccaaaatacataatttattcctaaatgttggtataatttgcagtttgaacgcttgaacaatttacgatggactgaattgataaccattgcttgtctaggatttgttgcaagctagtataaactattaaatgattgaataacgtttttacatcgaatttatgtgttcacgcttaaataaatcacagtttgagatgaaaatcttcaatattttcagttaattggaagaaatacttgattgtgtagatacgtaaaatgttttcacacaatttataattatgctgctacgttttgtttcatttattttactacaaaggaagaagacagacaataatttaatgcttactatgaatttttactaaactatttgcagtattacaaatcgtagtgatgtctgctgtcttatatacaacagaattggcaaaagaaaacgattcagaagcttcttattcaattacgtaaaaactagcaatagatctcccagaattttgtagctacacgtacagttctgggaataatatatgggcgtgggtctatctactttcgttttgatcaatgttaggaaaaaatgtgaggaacaacttcgaaaagaatatgaaatttcttcgctacaataaattttagatagaacaaattccttgagtttcttagaggtgctttctcaatgtatactaaatgattttaataactacaaatcttatacaaacatgaataaaaaaataatccaccgttccaattgaacagacaatttcaataaatcgatcctattgacttcaattagataactgcacagcatcatttacaaatcactactatttatataacttgaggcatttgacaaactggcctgatcgtgagcaggaggtcttaagtggagagagcaaggggcatcgacccgactgccttaactcaggttgtctgcggttgttagttcgtttgtgcgtttgtgcgcagctgcacttaacttgtgaatgcaataatagtctgaatgtactgaacatgcatggactgtgagatgaaaaaggattacgtaaagtcgaaacatgaagacgacgtttcatagtaatgtcagtcttatccataaaccgatagagtacataagcactcttcagaatgtgtgcatggtaaaaatgttaaaactatttccccaaaaactcttcccgaaaaacttatttcaatcaaaagctttgccgttaccgtaaaatttaatccgtaagcagatagaatacaatgcctcttacctttttcaacgcaagtgctattttgaactctcttaaaacgaggaaaagtcgattttaccttacgtgtgttggtaaaaagtgttctatattaagctatgtataaaattacactcatgtcgttggcaacccttgcttatattttgtgacggccctctggggacaagtcaatacataaggaaccggtcatgcgtgagtcgggattacgatgggtcccatatgttgtaaatatttttgagatcatcgtttactatgtagtgaaaggagatactatcatagagtgaaagcttcatctatatatgagaaggaaaatccgaaataagtggttaaagtcaatcggtagaatgtgctgacttctctaaccgtaaaagtaaagtctaatagaagactattctatccatttcttttttaattagatacgtaagtaattaatttgcagcgtttcgtagtactaaagcataaatattagtggagtgctatctaaacaattaaagacacattgattattaaaatcaaattcaaagcacaatcaatagatagcacgttgccttaatcccttaggagacagacaaagacaatcgcagacgctttcctgtgcctcatagaataactaaatacgacctacagcgacatttcctagaagaaataaattattcaacaacatacacaaatacttgcatacaaaatgcatcttcataacttactgtctgactaatatattttgaccgttgtgaagttaggcgcagtacagagagagcgggatgcagataagtacaaacgctagtgagtgtatgagacaacaatatacttgtagttaacttcagagagatcaagataaaatgtattgtattaataaagaacactaaaatgtatttttattacgttaaataagtatacgcacacaataaaacatttcttcagttgtctaatgtttgttttattattctaagatcgttggataaatataataattaagatgtataaattctttaggattgaatttgaatttcctttacgcattacactcataataataacataaacattcataactgtactgcagacgtgaatttatattagcctattatagtagagtgcaaaaacaaaataggagttattttattcacttgatatatattataaatgtaataagcggttttctagtcttacaataaaaattaatttggcaagtatatatttaaacgaaaactctatataagactaatgtaaccaaaccttaagcgtaaatctcatactagaaggtgtgttacatgtgacaattatcttacgtaggtaaatgagcaaatacttagtcatctgaacaaataaagctattttctgtaaaacagtcatcaaagtcaggtcgtaaataataagaactgaacacttagtatgacgcaattcccttgttgtgattttaaactatgtaggtacgaggtagataacggcatagcaaactattgttattcaacaatacctctttatgtatttatagactaaatataagttaaaatgaagtcaaaaaatctcttctattggtgagaatagccagtaacggatgtaactatacagtacccttagtatgagttttctttagatcaaacgaaaacgacgcgagagcgcgttcggcgctctgaatggtcgaaccatgaaccaaccaatcacagcgccgaacgcgctctcgttttgatctcgttaaacgtaaaacaaactcgtactaacccccctgacgatgattatgatactgatgataatgataacgcaacaactttctttacgaaaaattatattcgtacaacggaataaccatattgagataaccaatctaaaacaaacttgtatgtaggtaacactaagtgttctatagacggatatattattttcattttgcaaccgatcagccaagtagctgtgtatatgcataattataaaggttgaatagcttagatcatgtatttatttgcaacaatgttggtacatagagaccagtgcggacgcgataataatatttcactttcctcatgttcacgtcacctcttctttgatgttaagaactgtagcatttctagctaatagtgtgagaattagaactttagtactatcttgctttgtaagcttggaatctcaggaaattgtaatggcactgtttctagaccttgttgcgtactaaacattaggaaaaaaatgtaacaacaataattattcagaatttgaattgaaatataaagttaaagttaaataagttcattgctcttatttattagtgactactaaaataaatgtgtactaactgagtttttcaaagagattgtagttttatattttgtatattatttatcaaagcattagcttctgtttgcaattctatcatatttagtcaccaaaaattagaagatacttctgaacatttcttcgtggctggtcgctgtttacagaccacaattcgcattttaaaaatgtgtgtaactgtcaacgttaacttttactaagtacaagtaaaaataaaaaataaaaccgcaaagttacaaccggctacttcaaccgcaacttagaaactgtgtaactcattagtacttgtaatgtttatgatttattgtcaataggtttgtttatttacgatttgggcttagaacttgcttacaagtcttgtcactttgaacgttgacaggttttaattgtaaaaacaacgtaacaaatactaaaccaattcctttatctaaggtctttttggaccccaactctctttaacctctgaccatcaaatttaaactatatttttacaatgataaagttaatttgagattgattcagctctgtataactgtatttaataggtgaaatcaatttaccaacataattatcgacatgacaatcgcacattttcatcacaacatccaattttattcttgcatgaaatatccgattacaaataagacaattataaaattgtttctgcaattattcaatcaaacctgacatggtagttgggtgcctaatgaaatgaacaattgccttctgtttgcttattagttgatcgcttttgtaatcaacaattgaacggtgtctattgatctttattgctagatgtacccgcagttctcatgtactgtctccgatactttatgaagcgtttaattgtttgtaaggaagagaagtatataaagattacattgtgtgatgttttgtagtttctgccttctatagatatctatgtatatctgttaagttagaaaaagaattatgattctaataatgttcctttaagtttaaagctgatttattggactactaccttttttggcgcagatttaagacttaggtacgcctactcatacattattgttttgtgactttccaaatttatttgcgtgtggtttcttgaaatgcggatgcataccttaacggttatctatttagacaagtagcttgaatttatgcaattattgtttcgatacctaataacgattgctcacaaataacagtttaaaaagcacaattcctattccacgtattgtgttcttatcaaatgatcaaatctccccttttaataaaacacacctacataatgcatgttttttactacttagttaacagtcagttattcgggatgaataaatggtgacgtcacgaaggcgcgccgcccctggatcaaagatcgtttcgcttgaacacaccactatgcgtgcgcgcaactgctatagggctgccgtttcgtaaaaaacatcgaatacataatcaggagtgaaatacagaattgaatattaaataaatgcttcctgtttattaaaattcaaccttactaaacaaaaaaaactaagactatcgataatattagtttttactactgttttaaaaaatatgaattacgtacgaaaacgtttaccagcctattttcgtttactacatacatactgtcacaaagagttcaattgcgttacaacgaagttttacccaaattttatatcttgaggctttatcttattctcgaaaaacttacgaggccgtctcaaagacaaagtttcttgaagttgagagataaggaaaatatgtttgttacttccccttccttgttcccttactcactactcaagtatgcgtaaacctattgatacaaaacatagttctctactaagaaaataatattcaaactgctagaacagggcaaaggccaacttttctcgtggccttaagaagatccgtacacagatacctttataacaagtccacgactgtgacacatgccacgcataagatagcacataatttatatgtttagaaactaaagaatcagcgatattatctatttaagttggacgaattaaaattctggtcacgatttttagcattttcgttggtttgttttgagggccagccctgtctagaagccaatgccctatgagacagaacaacattgtgacgatttaccactcactcacaatgagcgaaaatagttctttccgacacgttcaagtatgatattcacaactacactcgagtaatgtaagcaataaatcacttagcactttgttgtacttaattttactaaacaaaacaaatcaaagactataaattacatacttacttaattaagtaacaacggtgtcaataggcgcttgttaatacgaattaaacactcctattctaatttaaaacatagcacgttaaattaattgcttgtaaactaacggaactcacgacctcatacattggatacttccataataaattataccgaaacaaaactagtttttatgcgttttagtgatttttgtcttgtagctagaatagtgcgtgccatctaatcatttcgtcataggatcaatttacactaatagtaagaaaagcatcaataatatcgttactcagttacggtgtcgattataacaatggatgcaagaaaatcgattgtcacgcgttgttattgtataatagttttatcatgcgatatgaaatgttgctttaatatggtcgttaaactttaggtaaacatgattttaatgatcatttttaagtaatatttttaaatgttttgagtatcattatgataacgttttttaacattcttaatacctatattcttgttagtcatgacgaaatactacagaaacacttcctcagctacaattagatcaaaagaagatttctcattgtattcaaaacagtgttccatatcaagaacttaagctcttaaagcgcaggaaactgcacatcacaacaacgcaaaacgtcaaacatcgatccactaacaataggagagagtctacatttgaatttcgagcacgaactattttaagatggcgtcccgaacgcacacgaaagtttacgacgattcgcgaattacgagccaataaagtgcggtgtcgccgacacgccgctacagcccttaaagcttttaatgtccatttaattgatataagctatacttaataatgctatcgatttcttttatagccggctttattgccagcttttaattatatgatccagttttatcttcaaattatttacacatacttgcatcactcaaacgcgctataaacataattccgtctcacgttaaagcatcatgcacctcggtaacgcaagacgtcttccgactatattttctgtcaccgagttaactgcacgacttccacaaatgtgagacagtgaccgagccttacttgttccataaataaccctcttactggacaaaaattctgttataattcacaatcagacctttaatatcccgatccgacgggtgggcattaatcaaatacatggaacgggtttgtacaaaggcaggcaagttaaataaaataataaataagctgcattccgtgagatttatttcgaagaaaaaaggctgtgtaagagccgggtcatgtcgcagtatgtttattgtccgcaccggtgtcggcccacaatgtacggctctaattaatgctatacctcgctctgagagatatatcgtgtccatccctgtgtctttttaagtcacgatcctctttttctaatctcgagattagtttccgacaatttattatttaaaaacacggacaacattaaaacatctttctgccctcaatactcttatcagattaggtcccaattacacaaaataatgcaaatgaggttgaagtactatatatatcctgtgcaggattagatttgattgttatcttaaacctatgggaacatacggtggttcccattaaaaaagatgagctcttacaggtacaatacgacgatagtcaaacctctccgcgacaacaatactgcagagaataatggaagccagctgacgggaggtgccttaacgcccgttgtcatggcgaccgtacgaagggtgaacctgcctaacatatcaccgaccccaacaaaaaactgccacgctcgcccttctggcatctgattttagactctaccccataaagcacaaggatcttttaacgcttaaccggacatctcttacgaccttttactttaatcgcattattgggatacgtgtattgtgttttgggttccatctttgctttttaaaattagaatgtctatcgctgtctcactctatctcacgaacactatctatccttgctcctctaaccacacttgtataattattccattgtctgtttatgacattttaaacagtttttaagtttttgttgaaataagcaccaaccataaatgttattgcggctgtaaaaatgcagtttcggtaatttatgggtcatgtaaacgaggtatcagagtttcttgcgattctcttgatttatacagtcactgcatattttttactttgtaatgagcgaagtgaaaaagttgtacgatatttaaatttttatatcactgaacataaattcacgttgttacataaaatattaggttacttcgtgccctcgtaaagaattaaacatttaaaagactttcattcaagcttactatggactcaatacaaaatttacattttaattaaacgattcgtttgtgtttgattaagaaaataccgatattatcgtcatcggaattaaataacgactacaaacttaatcgtatgcatttcaatacattatggaaagtgcttttacaaaattgtaatatgggaatgataaagtgcaaagaaattaagcgaacaaaatatttttaataaattaaattaatagataattatgacactcaccacacaatatctaggaatgataagaaattataataattgcttcattaaggttcgacatacctacttaattctatgcgcaacaatttaaagtgttggtacaccaattaaaataacccattagcagtatcgtatctgtttcttttaaagctatcaggtaactaaggccagcctacacggtcgagcattccgagacactgttggtccgctatcaattttatacacggtgggaaggctatttatcttaccttatttattggctgactcgaaaaccagtctcgggacccgatcgctttgtggaactcggtatttacctgtctatgtatactcgactttacgacgcgtatgttggatgagaatttgcctttcgtgaattcttaggtattacgattaatgtttaataaactgttaaatgtttaaagaattacgtatgagaactttacgacggtaaagggtaaactttcacgatttctaagaaaacgtcttaacagaaacgaaataaagggacaccttacaaagaattagaaagttgcacaacccttctttacgcatccctgagttttttgtagcgatttctttttagaaaggacactattgttcgcggaccgtttgcttctaaccctttccgttacttttttgcttcgcggcgatattgtttttacttattcttattgtaaatacaacagtacgacacaaacttagaacccgtacctacaaagacccaaagggcgcacgatttcgacgctctgtgattaatttccactgaggttggtctcagtagcggataactctaaaattaactttagtacgctataaaatattaatgagccattaaaatctaaaatggtataaaagtgaaatttaatggtttctctcgaagctttttgcgaatcgaagctgttggaagcagccgtgatatatgatgctataaatcgattttaaaaactccgattccaccgttacatttaatttatttcagattaaccttgtttttatgccgcatcagattaataaattgttaataaaactgaatgcataataatgattttctctttgtttcca***GGACCGAACGGATGTCCACGAAGGCGGGGCCGACAGACTTACACAAGGTTCCAAACGCTAGAACTAGAAAAGGAATTCCACTTCAACCACTATCTGACACGCCGACGCAGGATAGAGATAGCACACGCGCTCTGTCTTACAGAGAGACAGATCAAAATATGGTTCCAGAATCGACGCATGAAGTTAAAGAAAGAGCTGCGGGCGGTGAAGGAGATCAATGAGCAGGCCCGTCGAGACAGAGAGGAACAGGAGAGAATGAAGCAGCAACAGCAGGAGAAACAAGCCAAGCTGGAGAACCAACATCACGGGCACCACGTGACCCACCATCACGACCCGATGAAGATGCCCATCGACAAGGGCTCCAACGACCTACTCAAAGTGAACAAGGTCCCTACGTAA***GGTGTCAGCGACGCCGTATAGGAAATCTTGAC**T**GATTCTGTATCATATAGTGTGAGAGGAAGTGTTACGTCCATACGGACCTGCGGGCGTTAGTCGATTCGTATCCACTAGTTTTATGTTTTCGCGTCGGAGTCGTCCTCGTTGTGGAGAACGGCGTCCCGGTTTATGTAATAATGTTCCTTTAGTGCGAAGGACTTGCCTCGGTGTCGTTCGCTGGGTGGACAGTCTGGTGATGTGCTTTAAGCATTTATTTTGTTGCCATAACCACACCTTGTATTGCAATAATATTTTACTAAAATATACAAAAACGTGTTAGAATCGCTCTAAGGTTTATAGGAGCCCGAGTGAACTTCGGTATTGTTAGTTAAGATTTTAAATCTGTGAATCTCATAGTATTTATTGCTACCACTTACTCTTAATCCATTATGTTATATTTTTATTAATAGATATTATATTATGTACATTTTGATGTATATTTTATTATTTGTCCGCTTTTAGCGGACTGGTTATGTTTTGATGGTCACATTTTTTTATGATTGTAAAATTTGTTGTATTTTATCAGTTGATTTGGAAAGAGTTACTTTTTTATTTTTCTTTTTGACCAGATTGTGATTCACTTCAGTAGTTTTAGTACACAAATTGTTGTTTTGGTTTGATCCATTGGTTTCGATAGAATAATGTTATCGACAAATATTAAAATACCTGCATAGCTAATTCCATAAGAACTTAAAATGTATATTTATAACAAAATACGTATGGAAGTACAATTTCAAATAGATCTTCGTCGTAACGAGCGTTGTGTTTTATATTTTCGTCTTCAATAATTATGGATATTAAATGTAGACGGTATTTTTTGATAATTCTAGTTGAAAATACCTTGTCCATAAAGTAAATAGCAAACAAATAAGAACAAGATTATGGATCAGTACTGCACATGGCGTGTTGTCCATACATCCCAAGTCATCTGATGTTCAAAATTATATACGTAACGATGATACATATAATAAATTACAATAATGTGACTAAATATAAATTTTACACGTAATATTAAAAGTTGAGTAGTTTAAGTGTATTATACATAGGTAAATAGGTTCGACTTTATATGAAGTATTTGCTAAGACTAGGTACCTACAACATAAACCAATGTACGTCGGAATAAACAGTGGTCGCATTTTATCAGGCGAAAAGTCAATGTCTTCAAGTTCCTTTTTTTCAACGAAAGTGTTATAAAGGCACCGTTTCACGTACGAAACGGTGGCGAAATTATGTTTCCTGCAATTTTACGTAGCCATATGCACATTTGAATTTTCCAGCTCCGTAATTTCATACATAAAATACCTACAAAAGGTATTACTAGGAGCTAAGGAGCTCGGATGAAACTCAGAAATATACACGGACTTTTTTTCGCGAAAACGCGAGCTTGTTTAACATAGCTGGTTTACAAATTATTCCACACACCATATTCGCTTTCAATTCCACGTTGTGCCAAAATACATAATTAAAATAATTTATTAACACGAACATTGAAATATTTGTTCCGCGTCCAAAAACATGTTTTAAAAAATTCTTCGTTCTTCAACTCTATACGTAATAACTGAGCAAAATTAGAATTTTAAAAATTAGTCTTGATAGTCACCATTATACGTAAATTAATGTCAGGGTAGAATTAAAAAGGTAAATTAATAGATAACTGTATTAAAATTGGAATCTTCGCTTAATTTTTATAAAAAACACTATTTCTCTCTTTCGAAATCTGTCCACTGTGTTTTTTCTGAACAATGTATCTATAACTGTAGGCTCTGTAAAAGAAACATGAGTTTTATGTCGTGATATCATTAGAAGGGATTTAAGTCGTCGATGGATAAAAATTAATCGAGAAAACATGCAGAAATTATTATAAAAATAAAATACCGTCGAAATTTTTAAACGTTTGCCATTGAAACTTAAACGAGTTTACACAATTCATTAAAACAGTCAGTTGTAACATTAAAATGGCAATAAAACATATTCCACTTGTTTACCAAGGTTACTTAAAAAAAGGAAAATATTTACAATTTTAAAACAGGAAAATTTTATGTACAAAAACAAAAAAAATAATAAAATAGCCTTTAGAACTTGACACGTTTTCTCGATACAATCTCGACGACTTTAATACATTGGTTGTTCGTAACTAATAAATCTCTATAAATGTCGTGCACGTAACGCAATGGATACGTAGTTAGATTACGAGATTTTTGTATTGATTTCCATTAAACTTACTCTAGTGCGAGTGCAATAACTTCAAACCCACATTATTTCATTAAATTCGTAGTCCCGTTAAGATTGCGTACATATTAAGGTTGTCGTTAGTAATGTAACTTTATCCCATCTTATTTTTAAATATATATTTAAAAAATCGAGAAGGAAAATCGAACAAAATGTACTTAAGTAACTTCGATTTAAGTAGGCTTTATAAATTATAAAACGCATAGTTATTAAAATATCGTAGACACTTGTAATAAAAGTTAAGCGTAAATCCGATGATTAGTAATTGAATGAATAAAAAAGCGCTTAAAAATTATTTAGTATTGTTCGTAGATGGTCATTTGGAAGGTGAATGGAAAGCTTCTTTGTTAAGGGACGTACACACTGCAAAACTGATACGAGGTTGACATTTCATAACTATATGAGATGTGTATACGAATTAAATTGTTGTTTCTACATTTTGAGAACTAGTATTAGTTGTTTGGTCGGTTTTTGATTATTGTTTGGGACTGTGCAAATAGACAAGTTTGAAGTGTCAAGCATCTTCCTCTTAACTAAGTTTCGTATAACAATAGGTAAATCTTGTAACATAGTAATGATATTCGTGTGTATGTACGTCCGATGATTTATGTCAGGTACACTTTATTGTTTATGCCTCGTAAAGAACTGTAATCCACAGTGAAACAATGAGCAATTTGCTAAATAGATTATATATGTCCTCCATCCATAATGCTGTCTTGCCATATTGCTAGTGTTTTGTAGACTGTAGGTATTATACCTAACCGAACATTATATATCAATATTAATATTAATTAATGTATCATCTCACATTAGGTTATCACGTCTTTTCTCAATTTTATTCCAAAATTTTCGTCTTTACTTCGATTTGTCAAGTCTGTTATGTTATTTTTTAATCTTTGTTGCGTTGTCCAAGTTATAGTATTTTCATTTTTCTGTTTTCTTTTAATTTTACGATAACAAGACTTCCGTTTATTAGTTATTATTCTAAAAACGATCAATTTTTATCTTTAACATAAATGTAGCTATGTATCTATGTACCTATATCTATATTTTTGAATATAGTTATTAAATCGTCTTAAAGTTAAGTAACTGTAAATAGTCGTGAAAGTTTTACGTGGCGCATTATTTTTTGTTTATATTTTTTTGTTTTATGTTATGTATTTATGTATTGAGATGTGTCGTATGCCTGTGCAATTTGACCATGTTAAGGGATACCCGATACCTTGTAAGCACTATTGTGTGCAGGGTTCAAGATTATTTGGTCTTTTCGACATGGTTGTGAGTCGTAAGCGACTGTTACGAAAAGGTCTAATTGAATTGGATGTTGTGGAAATCGTTTTCATATCTGATGTAACTTGAACTGTAATTGAGTACACTGGTAGTCCAAGTTAACGTGGTCAAGTGTGAGCTAATGGTACGATTTAACGTGACTGTGTCGTGTTATATAAGCAAATAGTTGGGTGATTGTATATGAAGCCCGCATTCAATATTGTAATATTAATTTCAAAAATTCAAGAGAATAAAGAGTTAATAAAAGTATGGCTTTTTGATTTTTGCTGTGTAGAATCCCATGAACCATGATGTTTTATACTTAAGAATTATGAAATTAAATTACAAACATGAAGTACGTAAAAACATTTTAAATAAATTGAGTAGAAATGTACCTAATTCTATTACTGATATTTATAAAT
