# Supplementary figures and images for: CRISPR/Cas9 mediated knockout of the abdominal-A homeotic gene in fall armyworm moth (Spodoptera frugiperda)
Source: PLoS One. 2018 Dec 6;13(12):e0208647. doi: 10.1371/journal.pone.0208647 (PMC6283638; doi:10.1371/journal.pone.0208647)

**S3 Fig.**

Representative pictures of uninjected and *Sfabd-A* sgRNA-injected FAW embryos.


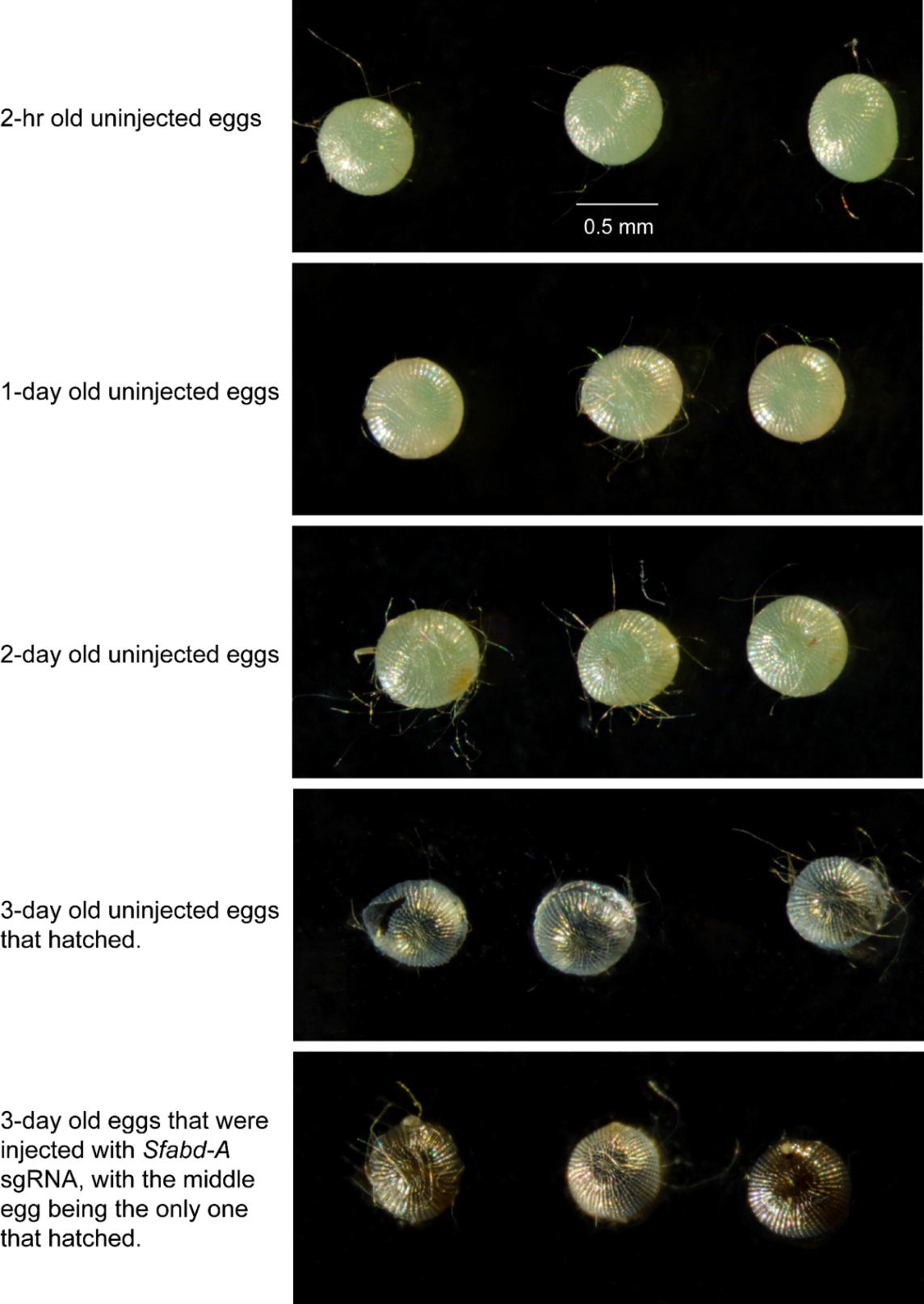

Supplement: S3 Fig — (DOCX) [file pone.0208647.s007.docx]
